# The dominant impact of dosing interval on the quality of T cells induced by SARS-CoV-2 mRNA and adenoviral vaccines

**DOI:** 10.1126/sciimmunol.adu4610

**Published:** 2025-08-29

**Authors:** Sam M. Murray, Ali Amini, Helen Ferry, Lucy C. Garner, Maria Fransiska Pudjohartono, Barbara Kronsteiner, Sagida Bibi, Andrew J. Pollard, Eleanor Barnes, Teresa Lambe, Susanna Dunachie, Paul Klenerman, Nicholas M. Provine

**Affiliations:** 1 Peter Medawar Building for Pathogen Research, Nuffield Department of Medicine, University of Oxford, Oxford, UK; 2 Translational Gastroenterology & Liver Unit, NDM – Experimental Medicine, University of Oxford, Oxford, UK; 3 Pandemic Sciences Institute, Nuffield Department of Medicine, University of Oxford, Oxford, UK; 4 Centre for Human Genetics, Nuffield Department of Medicine, University of Oxford, Oxford, UK; 5 NDM Centre for Global Health Research, Nuffield Department of Clinical Medicine, University of Oxford, Oxford, UK; 6 Oxford Vaccine Group, Department of Paediatrics, University of Oxford, Oxford, UK; 7 NIHR Oxford Biomedical Research Centre, University of Oxford, Oxford, UK; 8 Mahidol-Oxford Tropical Medicine Research Unit, Bangkok, Thailand

## Abstract

Functional T cell responses are crucial for protective immunity following COVID-19 vaccination, but factors influencing the quality of these responses are poorly understood. Here, we employ an activation induced marker (AIM) assay and single-cell transcriptomic sequencing to analyze SARS-CoV-2 spike-responsive T cells following mild SARS-CoV-2 infection or following one or two doses of mRNA-LNP or adenoviral vectored COVID-19 vaccines, administered at 3-4-week or 8-12-week dosing intervals. Our findings reveal broad functional and clonal T cell heterogeneity in T cells generated by vaccination or infection, including multiple distinct effector populations. T cell function was largely conserved between COVID-19 vaccine platforms but was distinct from T cells generated by SARS-CoV-2 infection. Notably, the dosing interval greatly influenced the quality of T cells after two vaccine doses, particularly after mRNA-LNP vaccination, where a longer interval led to reduced inflammatory signaling and improved secondary proliferation. These insights enhance our understanding of SARS-CoV-2 specific T cells and inform the optimization of mRNA vaccination regimens.

## Introduction

In addition to binding antibody titers (1), T cell responses are associated with protection from severe COVID-19 (2). However, compared with antibody responses, fewer studies have investigated T cell responses to leading COVID-19 mRNA-LNP and adenoviral vector vaccines encoding SARS-CoV-2 spike antigens. The complexity and heterogeneity of the T cell response following COVID-19 vaccination remains largely unexplored. While often referred to as a single aggregate response, T cell responses represent the activation of multiple heterogeneous cell subsets (i.e., CD8^+^, CD4^+^, T_H_1, T_H_2, T_H_17, T_FH_ and T_reg_) with diverse effector functions (e.g., cytokine production, cytotoxicity, cell-cell contact-dependent functions)(3). Thus, detailed studies are required to capture the multiple facets of an effective T cell response.

Enumeration of IFN-γ^+^ T cells by ELISpot or intracellular cytokine staining (ICS), is the most widely used measure of vaccine-induced T cell responses and has provided key insights into differences in the immunogenicity of vaccine platforms and in the intervals between vaccine doses. In randomized clinical trials (RCTs) ChAdOx1 nCoV-19 induced stronger T cell responses after the priming dose than BNT162b2, but two of three trials reported higher responses with BNT162b2 following a booster (i.e., second) vaccination (4–6). Observational studies have also shown higher magnitude T cell responses after one or two doses of adenovirus vector vaccines (ChAdOx1 or Ad26) compared with mRNA-LNP (BNT162b2 or mRNA-1273) vaccination (7–9). These studies indicate differences in the quantity of SARS-CoV-2 spike-specific T cells, but crucially lack insights into the quality or multi-faceted functionality of these T cells.

Along with vaccine platform, interval between vaccine doses has demonstrable impact on vaccine-induced T cell responses. To ensure maximum delivery of the first COVID-19 vaccine across the population and based on evidence of improved vaccine immunogenicity in early clinical trial data, the UK and Canada implemented an “extended” interval between first and second dose of mRNA-LNP vaccination. This increased the dosing-interval from the RCT-defined 3–4-week interval (short interval) to an 8–12-week interval (long interval). Both dosing intervals were found to provide high efficacy in population-level studies (10) and RCTs (11), with equivalent or increased efficacy in long compared with short dosing intervals in both vaccine types (12, 13). Regardless of vaccine type, RCTs and observational cohort studies showed that increased dosing interval marginally decreased the frequency of IFN-γ^+^ T cells (5, 14, 15). In contrast, using mRNA-LNP vaccines in an extended prime/boost dosing regimen markedly enhanced humoral immunity (5, 6, 14, 15), highlighting a disconnect between T cell and humoral immunity. Nevertheless, the frequency of IFN-γ^+^ T cells is only a single measure of cellular immunity, and further work is required to broaden these results to the full diversity of T cell functionality.

Some studies have begun to address this knowledge gap. Assessment of T cell function using multi-parameter ICS has demonstrated that ChAdOx1 and mRNA-LNP vaccines induce predominantly T_H_1-biased CD4^+^ T cell responses (4, 16). However, the ICS assay likely underestimates T cell responses and is limited by its ability to capture only specific cytokine-producing cells with relatively limited functional breadth. Activation-induced marker (AIM) assays have emerged as a new approach to quantify the frequency of antigen-specific CD4^+^ and CD8^+^ T cells and overcomes limitations in ICS by detecting T cells independent of their cytokine-producing functionality. In addition, the AIM assay can be used to sort live, antigen-responsive T cells for further analysis using flow cytometry. This can be done in a manner agnostic to HLA-type or precise *a priori* knowledge of specific target epitopes, challenges which limit the use of HLA-tetramers to sort antigen-specific T cells. In combination with transcriptomic analysis, the AIM assay may therefore be used to deeply phenotype polyclonal antigen-responsive T cells in various biological settings. This approach has previously been used to reveal lasting transcriptional differences in SARS-CoV-2 responsive T cells over 6-months after mRNA vaccination (17) and demonstrate skewing toward a hyper-effector phenotype after mRNA vaccination in XLA patients (18); highlighting its utility in profiling the functional diversity of antigen-responsive T cells.

In this work, we sought to broaden our understanding of how vaccine type and the interval between vaccine doses impacts on the functionality, transcriptional state, and diversity of CD4^+^ and CD8^+^ T cell responses induced by vaccination. To accomplish this, we performed multi-modal single-cell RNA, surface protein, and TCR sequencing on sorted AIM^+^ (“AIM-seq”) SARS-CoV-2 spike-reactive T cells using peripheral blood samples taken after one or two doses of BNT162b2 or ChAdOx1 nCoV-19 vaccination. To compare the observed vaccine-induced responses with immune responses following SARS-CoV-2 infection (ancestral strain), we sampled individuals after mild COVID-19 at two timepoints that were analogous to the post-dose one vaccine timepoint for the short or long interval. We show that AIM^+^ T cell populations induced after exposure to a SARS-CoV-2 spike antigen (through vaccination or infection) are functionally diverse including effector populations not usually identified, validating the power of an unbiased transcriptomic approach. While vaccine type had an unexpectedly nuanced impact on the phenotype and function of responding T cells, interval between doses had a more marked effect that was dependent on the vaccine platform. Collectively, these data provide new insights into the heterogeneity of the effector T cell responses generated by SARS-CoV-2 infection and vaccination and highlight the impact of dosing interval on T cell functionality.

## Results

### Study population

**Fig. 1:**
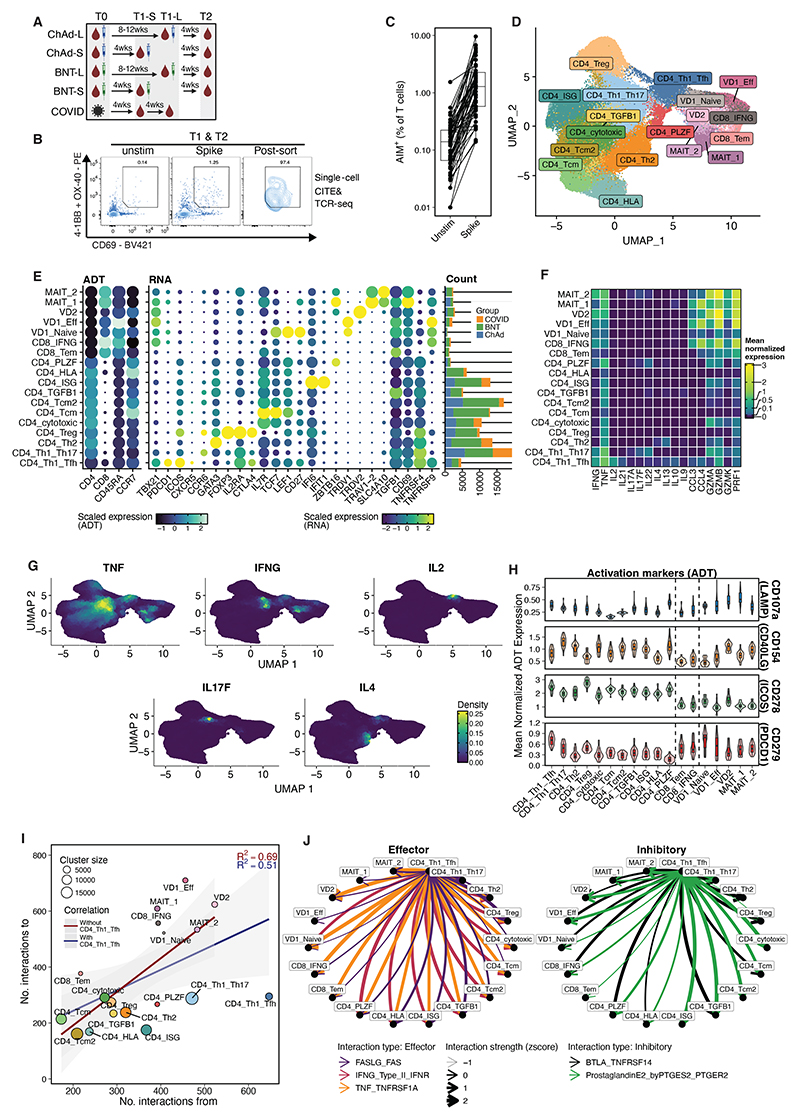
Identification of transcriptionally and functionally diverse conventional and unconventional T cells by AIM assay after SARS-CoV-2 spike stimulation. **A)** Study overview. **B)** Representative flow cytometric staining for sorting of SARS-CoV-2 spike peptide stimulated peripheral blood by activation induced markers (AIM). **C)** Proportion of AIM^+^ CD3^+^ T cells following 24h stimulation. **D)** UMAP representation of gene expression data from sorted AIM^+^ CD3^+^ T cells, with manual annotation. **E)** Selected surface protein (ADT) and RNA features used for manual annotation. **F)** Mean normalized RNA expression of selected functional genes. **G)** Projection of the RNA expression density of selected functional cytokines onto gene expression UMAP. **H)** Mean ADT expression of surface activation induced markers. **I)** Pearson correlation of total number of interactions to and from a given cell type. Point size is scaled by number of cells per cluster. Blue and red text is R^2^ with and without CD4_Th1_Tfh, respectively. **J)** Selected effector and inhibitory interactions that originate from the CD4_Th1_Tfh cluster. Arrow points in direction of interaction and is scaled by interaction strength.

To assess the impact of vaccine modality, number of vaccine doses given, and interval between doses on T cell phenotype and function, we sampled individuals with no history of symptomatic COVID-19 vaccinated with ChAdOx1 nCoV-19 (“ChAd”) or BNT162b2 (“BNT”) with a short or long dosing interval immediately before (“T1”) and 28-days after (“T2”) their second vaccine dose (Fig. 1A). Also included were a group of unvaccinated individuals with mild COVID-19, sampled approximately 28 days (range 17-28 days) and 56 days (range 53-61 days) after SARS-CoV-2 PCR positivity (“COVID”), corresponding to the T1 timepoint for the short and long vaccine groups respectively. Groups were broadly balanced for age and sex (Table S1).

To capture SARS-CoV-2 spike peptide-reactive T cell responses we used an activation-induced marker (AIM) assay (Fig. 1B). AIM markers were CD69 combined with OX-40 and/or 4-1BB. On average, 2% of CD3^+^ T cells were AIM^+^ (Fig. 1C), with little difference between study groups or timepoints (Fig. S1A). Median percentages of CD4^+^, CD8^+^ and CD4/CD8 double negative (DN) AIM^+^ T cells identified by flow cytometry were 86%, 7% and 6%, respectively (Fig. S1B). All AIM^+^ CD3^+^ T cells were isolated by flow cytometric cell sorting for multi-modal (RNA, TCR and eight selected cell-surface phenotyping and AIM protein markers) single-cell sequencing, with the largest numbers of AIM^+^ CD3^+^ T cells obtained from BNT vaccinees (Fig. S1C).

### AIM^+^ T cells elicited by COVID-19 vaccines display diverse phenotypes

After sequencing and QC, the final dataset of T1 and T2 AIM^+^ T cells contained 128,017 cells (Fig. 1D). Clustering and expert annotation (**Methods**) identified 18 clusters: 11 were identified as CD4^+^ T cells, two as CD8^+^ T cells, two as mucosal-associated invariant T (MAIT) cells, and three as γδT cells (Fig. 1E). All vaccine types, both timepoints and the majority (80%) of donors contributed to all clusters (Fig. S1D). CD4^+^ T cell clusters comprised multiple populations with clear parallels to known polarized subsets, including a hybrid T_H_1/T_FH_ cluster (CD4_Th1_Tfh), a hybrid T_H_1/T_H_17 cluster (CD4_Th1_Th17), a T_H_2 cluster (CD4_Th2), and a T_reg_ cluster (CD4_Treg) (Fig. 1D-F). Several clusters exhibited phenotypes not associated with T_H_ polarization, including a cytotoxic population (CD4_cytotoxic) and memory states (CD4_Tcm and CD4_Tcm2). Finally, four clusters of CD4^+^ T cells were defined by distinctive expression of specific markers (*TGFB1*, interferon stimulated genes [ISGs], HLA molecules, and *ZBTB16* [encodes PLZF], respectively) and could not be easily assigned to known CD4^+^ populations (CD4_TGFB1, CD4_ISG, CD4_HLA, and CD4_PLZF). The two clusters of CD8^+^ T cells corresponded to activated IFN-γ-producing effector CD8^+^ T cells (CD8_IFNG) and more quiescent effector memory T_EM_ cells (CD8_Tem) (Fig. 1D-F). There was strong positive correlation between flow cytometry and single-cell derived CD4, CD8 and unconventional T cell subsets (Fig. S1E). Flow cytometry-based phenotyping of AIM^+^ T cells aligned with the diverse T cell populations identified by AIM-seq (Fig. S2 and S3).

### Baseline AIM^+^ T cells have defined phenotypic and transcriptional characteristics

In the conventional AIM assay, background subtraction of the small fraction of T cells that are AIM^+^ at rest gives a very good signal-to-noise ratio (19–21). However, these cells cannot be excluded from sorting gates when isolating cells by flow cytometric cell sorting. Thus, we sought to identify sources of background in the AIM assay. Unstimulated PBMCs identified biased enrichment of specific T cell populations within the AIM^+^ population (Fig. S3). Notably, the background was impacted by duration of stimulation and many populations declined in frequency with 24-hours compared to 12-hours of incubation. Unconventional cells, and particularly Vδ2^+^ γδT cells, were particularly notable in this regard (Fig. S3C and S3D). Only AIM^+^ T_reg_s and naïve CD4^+^ T cells significantly increased in absolute frequency with extended incubation in the absence of stimulation. Interestingly, 24-hour spike peptide stimulation had minimal impact on the proportions of AIM^+^ T cells as compared to 12-hour stimulation, with the notable exception of increased T_reg_s and a trend towards decreased Vδ2^+^ γδT cells. Thus, 24-hour stimulation appears to effectively increase the signal-to-noise ratio primarily by reducing background activation.

To directly assign the transcriptional signatures identified in the single-cell RNA sequencing data with the baseline phenotype, we identified baseline AIM^+^ cells by surface expression of CD69, CD134 (OX-40), and CD137 (4-1BB) in a published CITE-seq dataset of unstimulated T cells prepared in a similar manner to our dataset (22) (**Methods**, Fig. S4A). Based on the original cluster annotations, MAIT cells were the population most over-represented in the baseline AIM^+^ population (Fig. S4B). When our AIM^+^ cluster labels were mapped onto the baseline AIM^+^ population, nearly all cells mapped to CD4_Tcm, CD8_Tem, VD2, and MAIT_1 cells (Fig. S4C). Critically, many of the polarized CD4^+^ T cell clusters we identified (e.g., T_H_1) did not appear to have a corresponding baseline AIM^+^ population, and were expanded compared to baseline, and therefore are distinct from AIM^+^ “background” T cell populations.

### AIM^+^ MAIT cells have signatures of cytokine-driven activation

MAIT cells cannot directly recognize peptide antigen (23, 24), but the frequency of AIM^+^ MAIT cells increased after 12- or 24-hour SARS-CoV-2 spike peptide stimulation (Fig. S3C). As evidenced by our analysis of baseline AIM^+^ T cells, their detection is at least partially attributable to their expression of AIM markers at rest. Baseline AIM^+^ MAIT cells only mapped to our MAIT_1 cluster, suggesting the MAIT_2 phenotype was activation induced (Fig. S4C). As we report in (Amini et al, co-submission), MAIT cells and γδT cells can be activated by IFN-γ released by peptide-specific T cells in a feed-forward cascade. To determine if this was detectable in the AIM assay, we first confirmed that the TCR characteristics of the MAIT_1 and MAIT_2 clusters mirrored those seen previously in sorted MAIT cells (25)(Fig. S5A-D). Clonal overlap was observed between the two clusters and across timepoints, suggesting the two clusters reflected plastic phenotypes (Fig. S5E). Strikingly, the MAIT_2 cluster had a signature of cytokine-mediated or dual TCR and cytokine (IL-12 and IL-18) stimulation, while the MAIT_1 cluster had a signature of TCR-driven activation (Fig. S5F and S5G). Thus, the MAIT_2 cluster appears to represent an effector population responding in a secondary manner to cytokines produced by antigen-specific T cells (Amini et al, co-submission), while the MAIT_1 cluster represents cells exhibiting residual in vivo activation.

### AIM^+^ T cells have diverse functional characteristics

To investigate how AIM^+^ T cells may relate to those captured by ICS assays, we next examined the production of an array of cytokines, chemokines, and cytotoxic effector molecules at T1/T2 (Fig. 1F and 1G). Canonical effector molecules of given T cell polarization states were produced as expected (Fig. 1F and 1G). *TNF* was broadly expressed and in some cases was the only detectable effector cytokine.

Different studies use different combinations of AIMs to identify peptide-reactive cells (19–21, 26). In addition to CD69, OX-40 and 4-1BB which were included in our staining panel, we performed CITE-seq staining for other commonly used AIM markers: CD107a, CD40L, ICOS and PD-1 (Fig. 1H). RNA transcript expression for CD69, OX-40 and 4-1BB was consistent with the expected protein-level expression for each AIM (Fig. 1E). Comparison of the surface protein expression of the other AIMs revealed interesting patterns between cell types and clusters, indicating that markedly distinct T cell populations can be recovered depending on the combination of AIMs used (Fig. 1H).

To better understand the function of AIM^+^ T cells, we examined putative cell-cell interactions between clusters, with the caveat that we could only examine T-T cell interactions in our dataset. Overall, CD4^+^ T cells sent and received proportionally fewer signals than CD8^+^ T cells and unconventional T cells (Fig. 1I and S6A). Classification of these interactions highlights diverse biology (Fig. S6B). The T_H_1/T_FH_ cluster sent the greatest number of signals to other clusters, suggesting a “hub” role for these cells (Fig. 1I and S6A). Examination of specific interactions revealed both immune stimulatory (e.g., IFN-γ, TNF and FASL interaction with their receptors) and immunomodulatory (e.g., BTLA and Prostaglandin E2 interaction with their receptors) interactions between the T_H_1/T_FH_ cluster and all other clusters (Fig. 1J).

### Antigen-specific and expanded clones are shared across T cell subsets

**Fig. 2:**
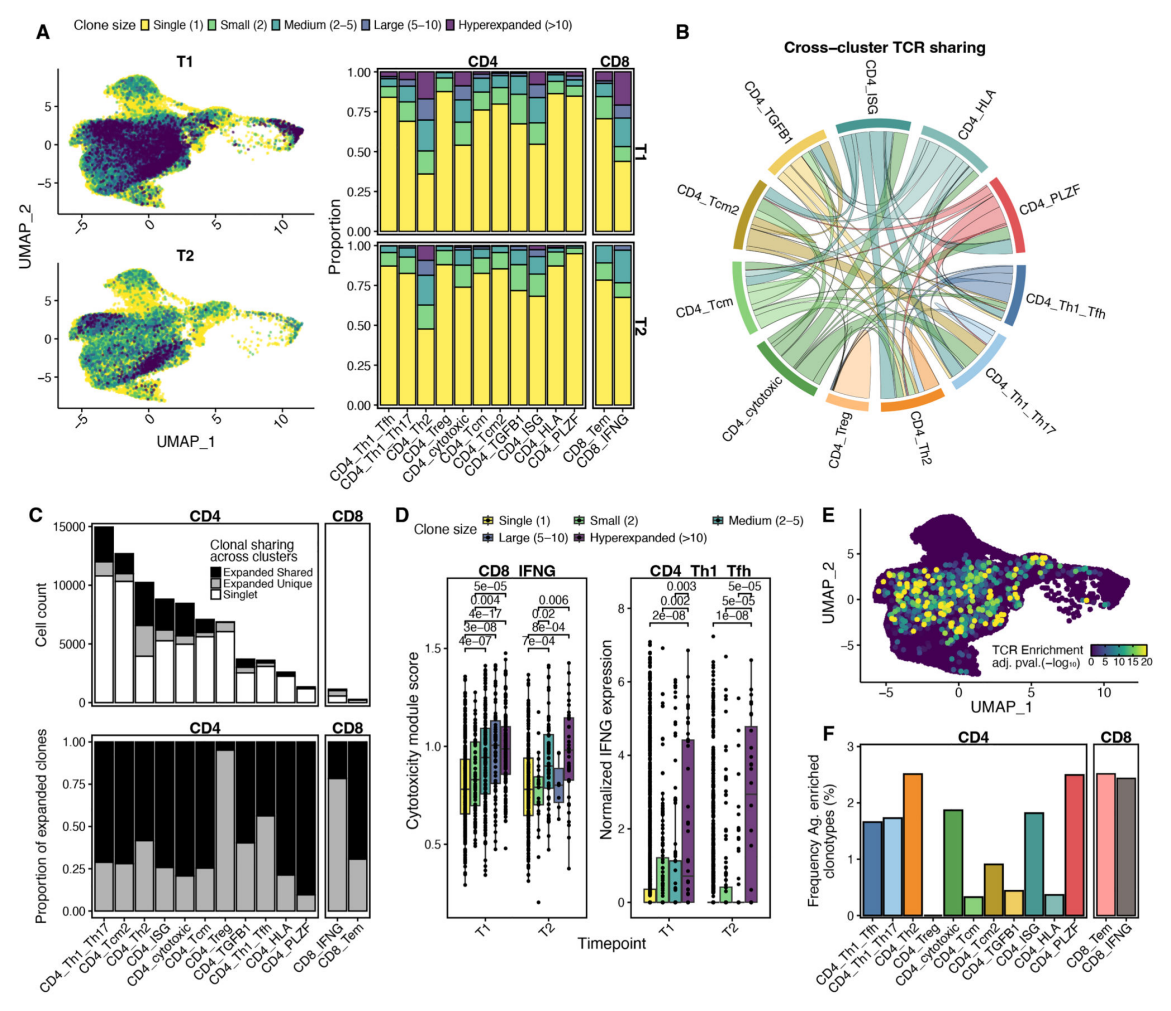
Expanded and spike-specific clones are found across AIM^+^ T cell subsets and are related to functionality. **A)** Proportion of cells within expanded or singlet clones per cluster and timepoint projected onto gene expression UMAP (left) or as proportion of each cluster (right). **B)** Sharing of expanded CD4^+^ T cell clones (clone size >1) across transcriptional clusters. Chord sizes reflect the relative proportion of each cluster that has shared clones. **C)** Number and proportion of clones that are singlets or expanded clones that are unique to a given cluster or shared across clusters. **D)** Per cell effector CD8^+^ cytotoxicity module score ((28), left) and effector CD4^+^ normalized *IFNG* transcript expression (right) as a function of clonal size (Mann-Whitney U test, Bonferroni adjusted). **E)** Gene expression UMAP colored by TCR meta-clonotype antigen-enrichment score p value (**Methods**). **F)** Frequency of clonotypes within each T cell cluster which are within an antigen-enriched TCR meta-clonotype (adjusted p value <0.05).

We next sought to determine the clonal relationship of the AIM^+^ CD4^+^ T cell populations. Clusters did not show specific enrichment of TCRα or TCRβ chains, with the exception of the CD4_PLZF cluster (Fig. S7A & S7B). Expanded clones were found across all clusters and at both timepoints, but were proportionally more abundant in T_H_2 cells, cytotoxic CD4^+^ T cells, ISG^hi^ CD4^+^ T cells and IFN-γ^+^ CD8^+^ T cells (Fig. 2A). There was considerable clonal sharing between CD4^+^ T cell clusters, with notable exception of the T_reg_ population (Fig. 2B and 2C). A major contribution to this clonal sharing was population interconversion between timepoints (Fig. S7C). Separate analyses of T1-long and T1-short timepoints demonstrated few differences in clonal sharing and expansion with time post SARS-CoV-2 exposure (by infection or vaccination) (Fig. S7D & S7E).

Excluding MAIT cells, CoNGA clonotype analysis (**Methods**) revealed that there was low overall correlation between TCR metric and gene expression when clonotypes of different cellular origins were aggregated based on their TCR sequence (Fig. S8A and S8B), reflecting the functional diversity of the captured clones. Despite this overall trend, one set of clonotypes within the IFN-γ^+^ CD8^+^ T cell population did have strong correlation between function and specificity (Fig. S8C). These clonotypes included one clone that matched a previously published SARS-CoV-2 spike specific clone (CASQETNTGELFF, (27)). In contrast to the CoNGA analysis, expanded clones within IFN-γ^+^ CD8^+^ and T_H_1/T_FH_ clusters were associated with increased cytotoxicity (published geneset from (28)) and *IFNG* production respectively (Fig. 2D). This analysis suggests that for a given effector state, functionality is associated with clonal expansion.

TCR meta-clonotype analysis (**Methods**) identified multiple antigen-enriched meta-clonotypes in both CD4^+^ and CD8^+^ T cell clusters and accounted for 0-2.5% of clonotypes within each conventional T cell population (Fig. 2E and 2F). Notably, none of the 6,001 unique clonotypes identified in the CD4^+^ T_reg_ cluster were predicted to belong to an antigen-enriched meta-clonotype (Fig. 2F). Furthermore, CD4^+^ T cells associated with the AIM^+^ background (CD4_Tcm, Fig. S5C) or with a more resting gene expression phenotype (CD4_TGFB1 and CD4_HLA) had lower frequencies of antigen-enriched clonotypes compared with other CD4^+^ T cell populations (Fig. 2F).

173 distinct meta-clonotypes were predicted to be antigen-enriched, including 11 with more than 10 unique participating clonotypes (Fig. S8D-G). The antigen-enriched meta-clonotypes included cells from several cell types (Fig. S8D). Interestingly, based on TCR gene usage, the largest antigen-enriched meta-clonotype matched the description of a CD1d-restricted invariant NKT TCR (29). This aligned with the CoNGA clonotype analysis, which independently identified clones within the PLZF^+^ CD4^+^ T cell cluster that were restricted to *TRBV4-1* gene usage (**Fig S8H)**. These TCR characteristics, combined with *PLZF* expression in this cluster, suggests that CD1c and CD1b auto-reactive CD4^+^ T cells may additionally be captured in this assay (30, 31). The presence of these cells in the AIM^+^ T cell population is likely caused by the same processes that result in identification of MAIT cells, as discussed above.

Other predicted antigen-enriched meta-clonotypes represent undescribed clusters of likely SARS-CoV-2 spike-specific T cell clones with similar TCR properties and are characterized in Fig. S8D-G.

### AIM^+^ T_reg_ cells are not antigen-specific but have an immune promoting phenotype

Our analysis suggested that the T_reg_ population was not a clonally-restricted antigen-specific population and detection of these cells was partially due to spontaneous activation after 24 hours of incubation without stimulation (Figs. 2 & S3A). The overall frequency of T_reg_ cells as a fraction of all CD3^+^ T cells did not change at 12 or 24 hours of incubation (Fig. S9A). However, AIM^+^ T cells were significantly enriched for T_reg_s in all stimulation conditions after 24 hours (Fig. S9A). A previously published scRNA-and TCR-seq dataset (32) of AIM^+^ T cells captured after stimulation of PBMCs from recent COVID-19 vaccinees with SARS-CoV-2 spike and CEF peptides also identified a large proportion of AIM^+^ T_reg_s to both peptide pools (Fig. S9B). Concordant with our data (Fig. 2B), re-analysis of this data identified minimal overlap of TCR clones between T_reg_ and CD4^+^ effector memory T cells for each stimulus (Fig. S9B). Strikingly however, there was comparatively more clonal overlap between T_reg_ populations induced by the different peptide pools (Fig. S9B). Together, this data suggests that AIM^+^ Tregs are spontaneously induced over the stimulation period, but their frequency is further increased by peptide in a non-antigen specific manner.

Despite their apparent lack of antigen-specificity, it is possible that T_reg_s play a role in regulating/maintaining the vaccine-induced response. Sub-clustering of spike-responsive T_reg_s identified multiple clusters with graded expression of CCR7, CD278 (ICOS), *LEF1* and *CTLA4*, suggesting the presence of both effector T_reg_ cells (CTLA-4^+^ ICOS^+^ FoxP3^int^ CD25^lo^) and memory T_reg_ cells (LEF1^+^ CCR7^+^ FoxP3^hi^ CD25^hi^) (Fig. S9C-E)(33). *FOXP3, IL2RA* and moderate levels of *IKZF2* (HELIOS) were expressed by all clusters, but there was minimum expression of T_reg_ effector cytokines *IL10, TGFB1, IL12A* and *EBI3* (Fig. S9D). In contrast to a suppressive response, the T_reg_ populations appeared to have an immune promoting phenotype. Multiple effector T_reg_ clusters had elevated expression of gene sets associated with antigen presentation (clusters 0-3) and positive regulation of inflammatory responses to antigen stimulation (cluster 3), compared with other T_reg_ populations (Fig. S9G). A putative reciprocal interaction between the T_reg_ cluster and nearly all populations involved cell-cell interaction via CD62L (*SELL*) binding to the receptor PSGL-1 (*SELPLG*). Additionally, T_reg_ cells uniquely produced IL-7 (*IL7* expression) (Fig. S9H).

### Pre-existing SARS-CoV-2-reactive T cells are expanded by COVID-19 vaccination

**Fig. 3:**
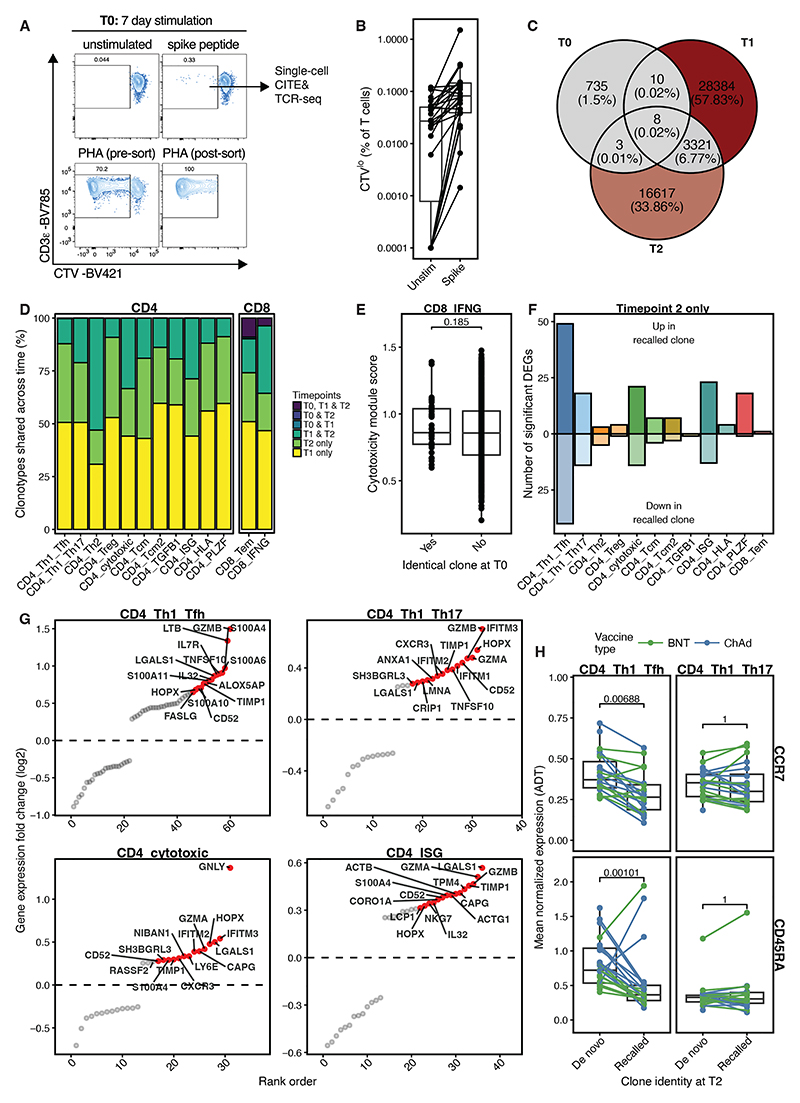
Recall of pre-existing or SARS-CoV-2 vaccine/infection induced T cell clones influences the function of AIM^+^ CD4^+^ T cells. **A)** Representative flow cytometric staining for sorting of SARS-CoV-2 spike peptide stimulated peripheral blood by cell trace violet (CTV). Positive control (phytohemagglutinin; PHA) plot also shown. **B)** Frequency of CTV^lo^ T cells after 7 days with (spike) or without (unstim) SARS-CoV-2 peptide stimulation. **C)** Number of unique paired αβ TCR clones that are shared across study timepoints in conventional spike-responsive T cells. **D)** Proportion of post-vaccine spike-responsive conventional T cell clones that are shared across timepoints within an individual donor. **E)** Cytotoxicity module score (28) of post-vaccination effector CD8^+^ cells that shared or did not share clonality with a pre-vaccination spike-responsive T cell (students T-test). **F)** Number of within-cluster differentially expressed genes (DEGs, average log_2_ fold change >0.25 and adjusted p <0.05) between post-second dose (T2) T cells with (recalled) or without (de novo) a shared identical paired TCR at an earlier study timepoint. **G)** Significantly differentially expressed genes (as in F) from selected CD4^+^ T cell clusters with highest frequencies of DEGs. **H)** Surface protein (ADT) expression of T cell memory markers in selected CD4^+^ T cell clusters at T2 (Mann-Whitney U test, Bonferroni adjusted).

Pre-existing SARS-CoV-2 cross-reactive T cells are well described (34), but the extent to which these cells contribute to vaccine-induced immunity remains unclear. To address this, we used a seven-day cell proliferation assay at the pre-vaccine timepoint (“T0”) to identify spike-specific T cells in unexposed individuals (35) (Fig. 3A and 3B). After QC, we recovered 1,050 cells comprising CD4^+^ and CD8^+^ T cell populations with naïve and effector phenotypes (Fig. S10A-D). Surprisingly, unconventional T cells were also recovered (Fig. S10A-E). Reference mapping the T0 dataset to the T1/T2 AIM dataset found only a portion of clusters described in the T0 data, with logical concordance between annotations (Fig. S10F).

From the T0 timepoint we identified 756 unique paired TCR clonotypes. Only 21 (2.8%) of these overlapped with either of the post-vaccine timepoints and only eight (1.1%) were found at both timepoints (Fig. 3C). Strikingly, pre-existing spike-reactive T cells were found post-vaccination only in the BNT group (0 out of 22,368 cells in pre-existing clones ChAd vs 94 out of 40,569 cells in pre-existing clones BNT, p = 1.46×10^-18^, Fisher’s exact test) (Fig. S10G). Baseline clones identified at post-vaccine timepoints were mostly found in CD8^+^ T cell clusters (Fig. 3D). Post-vaccination, pre-existing clonotypes were not more cytotoxic than ones only identified after vaccination (Fig. 3E). Collectively, these data suggest that pre-existing cross-reactive T cells make only a minor contribution to the overall vaccine-induced response.

We next examined if clones induced by the primary vaccine and recalled by the second dose (detected at T1 and T2; “recalled clones”) were different from clones only detected at T2 (and thus more likely to be a *de novo* response of vaccine dose 2). Differential expression analysis revealed a limited set of genes in each cluster between recalled and *de novo* clones, with the largest number of differences in the T_H_1/T_FH_, T_H_1/T_H_17, cytotoxic and ISG^hi^ CD4^+^ T cell clusters (Fig. 3F). *SLAMF1* was the only significantly differentially expressed gene between recalled and *de novo* clones in IFNγ^+^ CD8^+^ T cells. Cytolytic components such as *GZMA, GZMB* (Granzyme A & B), *GNLY* and *LGALS1* were amongst the most upregulated genes in recalled versus *de novo* clones in CD4^+^ populations (Fig. 3G). In T_H_1/T_FH_ cells but not T_H_1/T_H_17 cells, this corresponded with a more effector-like phenotype, with decreased protein expression of CCR7 and CD45RA (Fig. 3H). Thus, effector function and memory phenotype differ across recalled and *de novo* clones in CD4^+^ populations, but not in CD8^+^ T cells.

### Distinct T cell responses are induced by ChAdOx1 nCoV-19 and BNT162b2 vaccines

**Fig. 4:**
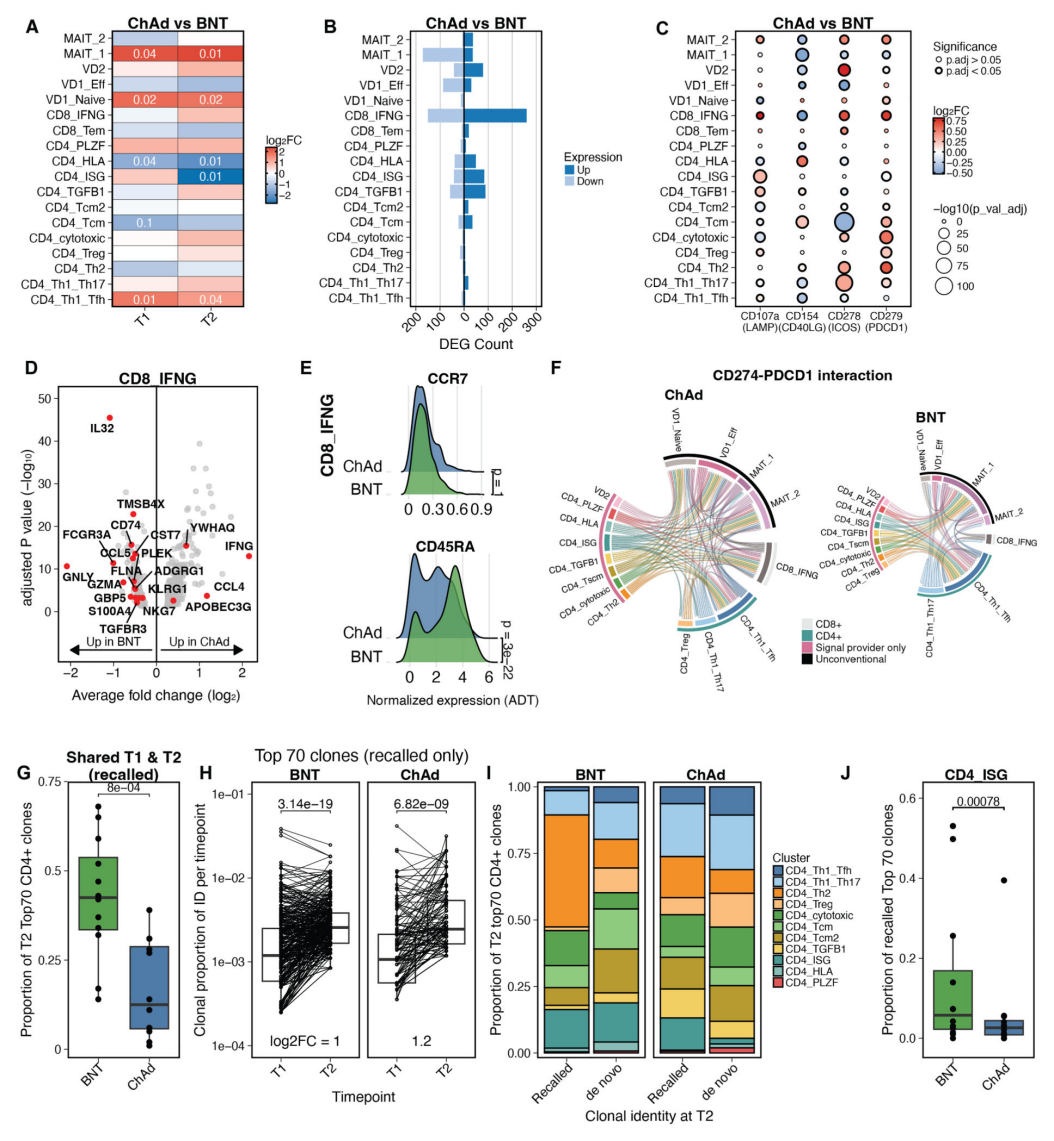
BNT162b2 and ChAdOx1 nCoV-19 vaccine vectors induce functionally distinct SARS-CoV-2 spike-responsive T cells. **A)** Log_2_ fold change (FC) in relative abundance of spike-responsive T cell clusters at pre- (T1) and post- (T2) second vaccine timepoints. Benjamini-Hochberg derived false discovery rate (FDR) values for comparisons with an FDR ≤ 0.1 are denoted. **B)** Number of significantly differentially expressed genes with an average log_2_ FC >0.25 and adjusted p <0.05 between ChAd and BNT vaccinees at T1 and T2. **C)** Average log_2_ FC of normalized activation induced marker surface protein (ADT) expression. **D)** Average log_2_ FC in normalized gene expression between spike-responsive IFNγ^+^ CD8^+^ effector T cells of ChAd and BNT vaccinees. Labelled genes are those included within a cytotoxicity-associated geneset *(28)*. **E)** Normalized expression of selected surface proteins within spike-responsive IFNγ^+^ CD8^+^ effector T cells. **F)** Number of significant *CD274-PDCD1* interactions between each cell type. Size of plot is scaled to total number of significant interactions, the arrow and distance of the chord end to the circle edge denotes the directionality of the interaction. **G)** Proportion of the top 70 largest paired CD4^+^ T cell clones at T2 that share an identical clone at the T1 timepoint (recalled T cell). Mann-Whitney U test, Bonferroni adjusted. **H)** Proportion of total cells per individual/timepoint of top 70 largest clones (Paired t-test, Bonferroni adjusted). **I)** Transcriptional cluster phenotype of recalled clones, or clones which are only found at T2 (de novo) within the top 70 CD4^+^ T cell clones. **J)** Proportion of recalled top 70 clones that have a CD4_ISG phenotype at T2. EdgeR comparison (Methods). BNT, BNT162b2 vaccinees; ChAd, ChAdOx1 nCoV-19 vaccinees.

Having described the AIM^+^ T cells as a complete dataset, we next examined the impact of vaccine type on activated T cell functionality. ChAd vaccination induced a significantly greater proportion of T_H_1/T_FH_ CD4^+^ T cells at both the T1 and T2 timepoints compared with BNT, in addition to select unconventional T cell populations (MAIT_1 and naive Vδ1^+^ γδT cell clusters) (Fig. 4A). Additionally, the ratio of MAIT_2 (cytokine- or TCR+cytokine-stimulated) to MAIT_1 (TCR-stimulated) and effector Vδ1^+^ γδT to naive Vδ1^+^ γδT cells was higher in BNT compared with ChAd (Fig. S11A and S11B).

Differential gene expression analysis per cluster revealed only minor differences between ChAd and BNT vaccination (Fig. 4B) that were largely consistent across timepoints (Fig. S11C). Differential expression of AIMs at the protein level were also observed between groups. CD107a (*LAMP*), ICOS and PD-1 (*PDCD1*) expression was significantly increased in the IFN-γ^+^ CD8^+^ T cell population in ChAd compared with BNT (Fig. 4C).

There were specific gene expression differences of interest between vaccine groups, particularly in the IFN-γ^+^ CD8^+^ T cell cluster. Examination of a signature of cytotoxic function found discordant differential expression based on vaccine type (Fig. 4D). *IFNG* expression was significantly higher in response to ChAd vaccination, while BNT induced significantly greater levels of cytotoxic granule molecules (*GZMA* and *GNLY*) (Fig. 4D). This was largely consistent between timepoints, however several ISGs were identified which were only upregulated in BNT vs ChAd vaccination at the T2 timepoint (Fig. S11D). These differences corresponded to a more T_EMRA_ phenotype in CD8^+^ T cells induced by BNT compared with a mixed T_EM_/T_EMRA_ phenotype induced by ChAd (Fig. 4E & S11E). Comparison of putative cell-cell interactions between vaccine types indicated that the majority of signaling pathways were shared by vaccine type (Fig. S11F), with a small number of interesting differences. CD160, a co-inhibitory receptor (36), was uniquely expressed by IFN-γ^+^ CD8^+^ T cells and effector Vδ1^+^ γδT cells from ChAd vaccinated individuals (Fig. S11G). Interaction with its receptor HVEM (*TNFRSF14*), expressed on all clusters, represented a broad and distinct feedback mechanism (Table S2). PD-L1/PD-1 signaling differed based on vaccine type, with a greater number of involved cell types and cell-cell interactions in ChAd vaccination relative to BNT (Fig. 4F).

Finally, we examined the impact of vaccine type on TCR usage. A substantially larger proportion of the top expanded CD4^+^ clonotypes at T2 were also identified at T1 following BNT compared with ChAd vaccination (Fig. 4G). Amongst the top expanded clonotypes at T2, clonotypes found at both timepoints (i.e. “recalled” clones) significantly increased as a proportion of total cells at T2 compared with T1 within BNT and ChAd vaccinees (Fig. 4H). Across all clones in vaccinees, clones that were recalled at T2 were proportionally larger and more expanded than non-recalled clones at T1 (Fig. S11H & S11I). Compared with top clones only found at T2 (“*de novo”* clones), there was an increased proportion of clones with a T_H_2 phenotype in the recalled clones in individuals in both vaccine groups (Bonferroni adjusted p<0.001, Fisher’s exact test) (Fig. 4I). Notably, there was a significantly higher proportion of CD4^+^ T cells with an ISG^hi^ phenotype in top recalled clones in BNT compared with ChAd vaccinees (Fig. 4J), highlighting the important contribution of this interferon-driven cell type to the recall response, singularly in BNT vaccination.

### Different dynamics of AIM^+^ T cell response induced by COVID-19 vaccines and SARS-CoV-2 infection

We next compared the phenotype of AIM^+^ T cells induced at early and late timepoints post SARS-CoV-2 infection and at equivalent timepoints after a priming vaccine dose. There were significant differences in cell composition between vaccinees and COVID-19 convalescent individuals that were largely consistent at both T1-long and T1-short timepoints (Fig. S12A), but T_H_1/T_FH_, T_H_1/T_H_17 and the IFN-γ^+^ CD8^+^ T did not differ in proportion. Compared with difference between vaccine vectors (Fig. 4B) there were considerably more differentially expressed genes between vaccinees and COVID-19 convalescent individuals, particularly at the T1-short timepoint (Fig. S12B). There was significant positive enrichment of genesets related to IFN-γ, TNF, and IL2 signaling in COVID-19 convalescent individuals at the T1-short timepoint, which decreased over time and was reduced compared to vaccine induced responses at the T1-long timepoint (Fig. S12C). These data suggest differences in early effector responses driven by infection versus vaccination which transitions towards a more convergent phenotype over time.

### Time interval between vaccine dose 1 and dose 2 impacts on the T cell response

We next examined the impact of dose number on the vaccine-induced T cell response. Despite the boosting dose, a comparison of T1 versus T2 revealed only minor differences in the relative abundance of different cell populations (Fig. S13A). TGFB1^+^ CD4^+^ and Vδ2^+^ γδT cells decreased at T2 compared with T1 in BNT vaccinees, but no significant differences were observed between timepoints in ChAd vaccinees. Correspondingly, few genes were differentially expressed by timepoint, and increased gene expression at T2 compared with T1 was primarily associated with BNT vaccination (Fig. S13B).

**Fig. 5:**
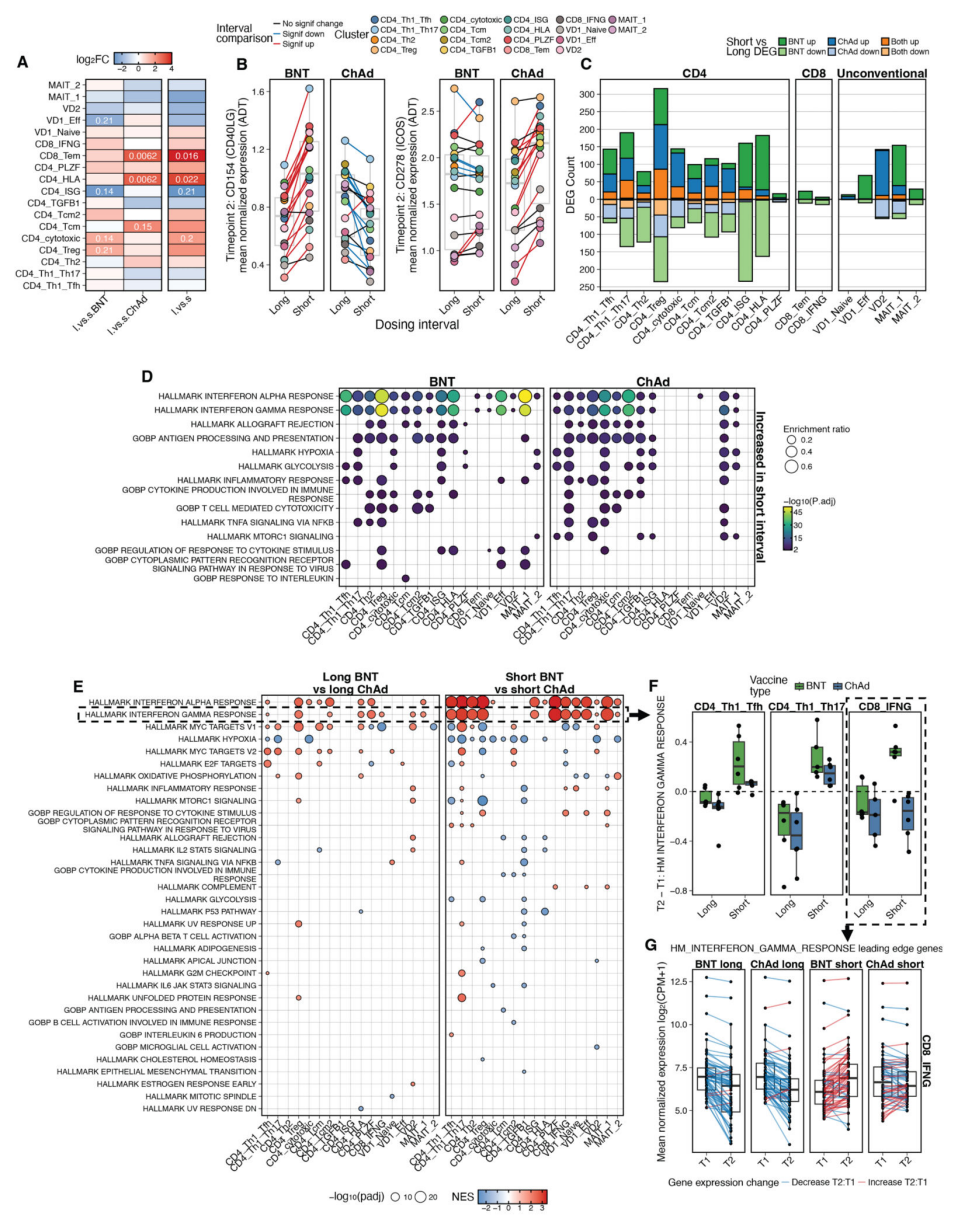
Dosing interval differentially impacts BNT162b2 and ChAdOx1 nCoV-19 induced spike-responsive T cells. **A)** Log_2_ fold change (FC) of post-second vaccine (T2) spike-responsive T cell clusters. Benjamini-Hochberg false discovery rate (FDR) values for comparisons with an unadjusted p value <0.25 are denoted. l, long interval; s, short interval. **B)** Mean normalized surface protein expression (ADT) of activation induced markers in T2 spike-responsive T cell clusters (signif = Bonferroni adjusted p <0.05). **C)** Number of significantly differentially expressed genes (DEG) with an average log_2_ fold change >0.25 and adjusted p <0.05 within T2 spike-responsive T cell clusters. **D)** Overrepresentation of genesets in upregulated genes identified in panel C. **E)** Enrichment of genesets in genes ranked based on the strength of association of their expression with the given comparison (**Methods**) at T2. Only enrichments with adjusted p value <0.01 are shown. **F)** Difference between T2 and T1 in the aggregate expression of genes within the Hallmark Interferon Gamma Response (M5913) geneset in selected spike-responsive T cell subsets. **G)** Mean normalized expression of genes within the Hallmark Interferon Gamma Response geneset in spike-responsive CD8^+^ effector T cells. BNT, BNT162b2 vaccinees; ChAd, ChAdOx1 nCoV-19 vaccinees.

However, when T2 was separated based on the interval between first and second vaccine dose, substantial differences were observed – many of these were in opposite directions based on interval and thus masked the observations when T2 was analyzed in aggregate (Fig. 5A). The long interval was associated with increased abundance of T_EM_ CD8^+^ T cells and HLA^hi^ CD4^+^ T cells in ChAd vaccinees, a phenomenon not observed in BNT vaccinees (Fig. 5A).

Across clusters, surface effector molecule expression was differentially impacted by vaccine type and interval. CD154 (CD40L) was significantly upregulated in short compared with long BNT, but the opposite was seen for ChAd. This pattern was reversed with CD278 (ICOS), which was upregulated specifically in short ChAd (Fig. 5B). Differential gene expression analysis revealed major differences based on interval for both the CD4^+^ T cell and unconventional T cell clusters (Fig. 5C). Strikingly most of these genes were unique to ChAd or BNT, with relatively little overlap.

Overrepresentation analysis highlighted broad differences in the strength of geneset enrichment across the clusters in short versus long interval for both BNT and ChAd (Fig. 5D). Genesets related to IFNa and IFNg were more significantly overrepresented in multiple cell types in the short BNT group but less so in short ChAd; whereas processes related to mTOR signaling, hypoxia and glycolysis were more strongly increased in short compared with long ChAd (Fig. 5D). Conversely, when assessing long interval boosting for either vaccine platform, relatively few annotated biologic processes were enriched for the upregulated genes (Fig. S13C).

Based on these findings, short interval and long interval were compared head-to-head for the two vaccines. GSEA analysis identified few differences in the cell processes or signaling pathways induced by long BNT versus long ChAd (Fig. 5E). In contrast, short BNT more strongly induced type I and II interferon signatures across the majority of cell types compared with short ChAd (Fig. 5E). Examining this in detail in the IFN-γ-producing conventional T cell clusters revealed different patterns based on cell type (Fig. 5F). For the T_H_1/T_FH_ and T_H_1/T_H_17 CD4^+^ T cell clusters, there was stronger induction of this pathway in both vaccine types by the short interval and downregulation in the long interval, but it was more strongly induced in short BNT compared with short ChAd (Fig. 5F). For IFN-γ^+^ CD8^+^ T cells, it was uniquely induced by short BNT at T2, with decreased signaling for all other regimens at T2 relative to T1 (Fig. 5F). This could be seen as coordinated induction of genes in this biologic pathway specifically in this vaccine condition (Fig. 5G). In contrast, a hypoxia pathway, known to regulate effector T cell function (37), was more strongly induced in nearly all CD4^+^ T cell clusters in short ChAd relative to short BNT (Fig. 5E). Together, these data highlight the critical role that interval between first and second dose of vaccine has on resultant T cell functionality, and that the biology is not concordant between vaccine types.

### Short dosing interval of BNT162b2 induces a more inflammatory recall T cell phenotype

**Fig. 6:**
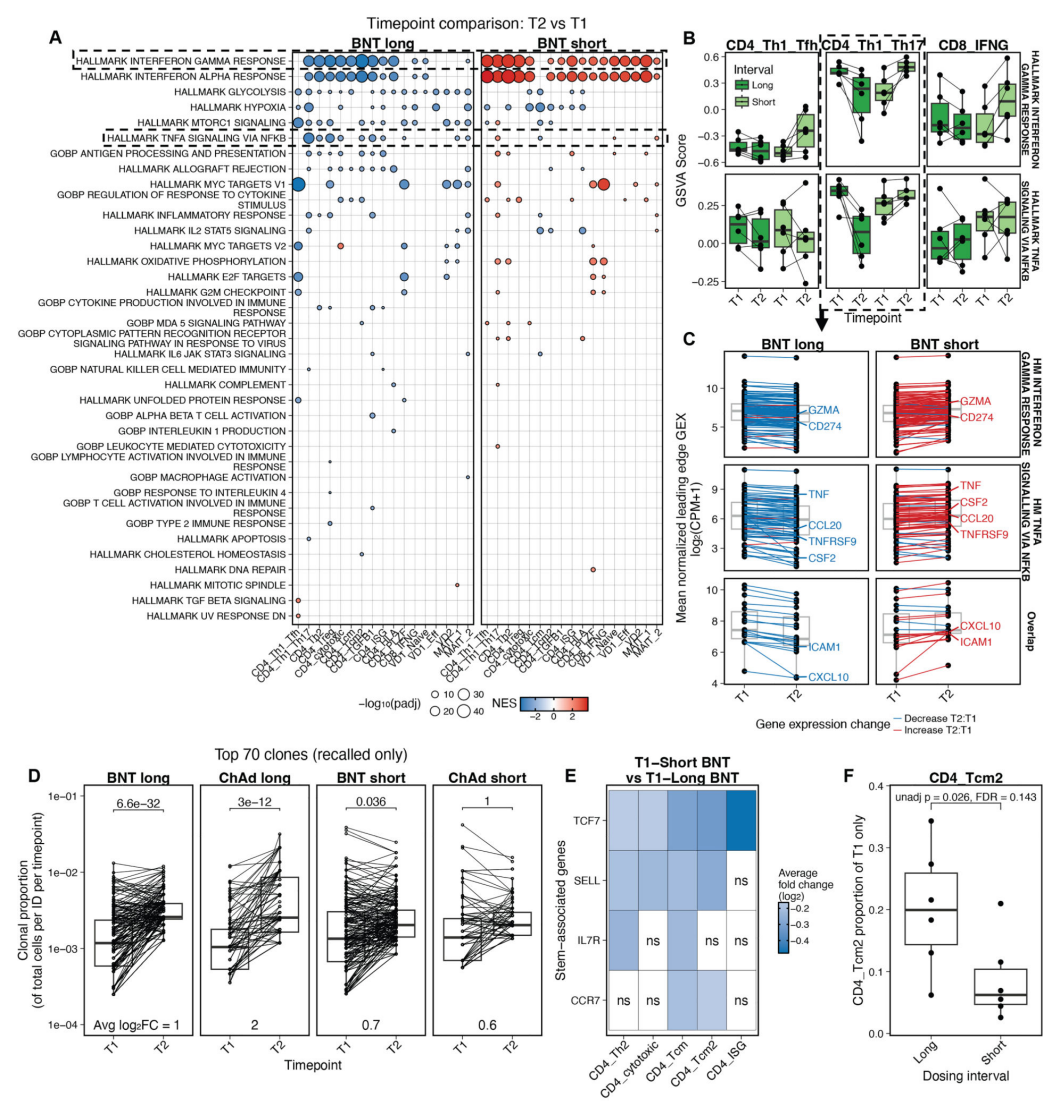
An extended BNT162b2 vaccine dosing interval induces spike-responsive T with reduced inflammatory functionality. **A)** Enrichment of genesets in genes ranked based on the strength association of their expression with the given comparison (**Methods**). Comparisons are made between paired timepoints, controlling for variation across individuals. Only enrichments with adjusted p value <0.01 are shown. **B)** Geneset variation analysis (GSVA) scoring of aggregate expression of genes within the Hallmark Interferon Gamma Response (M5913) and Hallmark TNFA signaling via NFKB (M5890) genesets. **C)** Mean normalized expression of genes within the Hallmark Interferon Gamma Response geneset in spike-responsive CD4^+^ T_H_1/T_H_17 effector T cells. **D)** Proportion of total cells per individual/timepoint of each clone that is shared within an individual at T2 and T1 (recalled) and within the top 70 largest clones at T2 (Paired t-test, Bonferroni adjusted). **E)** Average log_2_ fold change of gene expression of selected stem-associated genes. Genes with log_2_ fold change <0.25 or adjusted p >0.05 denoted by ns. **F)** Proportion of T1 AIM^+^ T cells represented by CD4_Tcm2, a stem-associated T cell cluster. Unadjusted p value and false discovery rate (FDR) presented. EdgeR comparison (Methods). BNT, BNT162b2 vaccinees; ChAd, ChAdOx1 nCoV-19 vaccinees.

A particular characteristic of the short interval is that the time since last dose at T1 and T2 is the same (28 days). Thus, we sought to determine how the T cell phenotype changed in the BNT group between first and second dose depending on the interval. In the short but not long BNT interval, type I and II IFN signaling were elevated across all cell types at T2 relative to T1 (Fig. 6A and 6B). The short interval between doses also led to sustained TNF signaling between T1 and T2, while the long dose resulted in reduced induction of this pathway post-boost (Fig. 6A and 6B). At an individual gene level, this reflected coordinated changes in expression of nearly all genes in the signature, with little overlap of the two signatures, suggesting multiple parallel inflammatory pathways (Fig. 6C). There were no differences between the phenotypes of AIM^+^ T cells in unstimulated samples in long compared with short interval vaccinees at T1, implying no differences in baseline vaccine associated bystander activation (Fig. S14A and S14B). Unlike BNT vaccination, the short dosing interval in ChAd vaccinees induced a less dramatic IFN and TNF response at T2 relative to T1 (Fig. S14C).

We next investigated the impact of vaccine dosing interval on the expansion and recall of TCR clones. There was less expansion of recalled clones in individuals vaccinated with a short dosing interval of either vaccine (Fig. 6D). Overall, across multiple clusters, there was evidence of reduced activation and inflammatory signaling at T2 in long BNT compared to T1, which may be associated with preferential recall of secondary (“recalled”) effector cells, while most of these signatures were equally robust (or higher) at T2 in the short BNT group. Thus, increasing the time between doses of mRNA vaccines results in a secondary response that is less inflammatory with lower production of effector molecules, such as *TNF, GZMA, CCL20, CXCL10* (Fig. 6C) and a larger average expansion of recalled clones. Compared to the short-dosing interval, the long-dosing interval was associated with increased expression of stem-cell associated T cell genes in multiple AIM^+^ T cell clusters (Fig. 6E) and enrichment for AIM^+^ central memory phenotype cells (CD4_Tcm2) (Fig. 6F) immediately pre-second vaccine (e.g., T1).

## Discussion

In this study we sought to determine how ChAdOx1 and mRNA (BNT162b2) vaccine platforms, number of doses, and interval between vaccines impacts the phenotype, functionality, and clonality of spike-specific T cell populations. To accomplish this, we utilized an AIM assay combined with scRNA-seq and scTCR-seq (“AIM-seq”) to gain insight into antigen-specific T cells independent of cytokine production functionality. Validating the use of this unbiased approach, peptide responsive T cells included not only the expected IFN-γ^+^ CD4^+^ and CD8^+^ T cells but also CD4^+^ T_H_2 and CD4^+^ T_H_17 cells, in addition to other diverse subpopulations. Interval between doses had the largest impact on T cell phenotype and function, with a short 3–4-week interval between doses resulting in a markedly more pro-inflammatory T cell response induced by BNT162b2 vaccination, and to a lesser extent ChAdOx1 vaccination. These data reveal unexpected functional heterogeneity in vaccine-induced T cell responses and a significant impact of the interaction between vaccine type and dosing interval on T cell function.

Comprehensive analysis of CD4^+^ T cell responses is complicated by both functional heterogeneity and HLA genetic diversity, which limits the use of ICS and MHC tetramer-based approaches (38, 39). The AIM assay is appealing because it identifies cells responsive to a specific antigen in a function-independent and HLA-agnostic manner. The most pressing issue to generate robust data from AIM-seq is that background AIM^+^ T cells cannot be removed (i.e., background subtraction) if cells are being isolated by flow cytometric cell sorting for sequencing. Thus, understanding the phenotype of contaminating cells is critical for subsequent data interpretation.

Flow cytometric assessment and reanalysis of published CITE-seq data (22) revealed that “background” cells are associated with very specific phenotypic/transcriptional states, including CD4^+^ T_CM_ cells, CD4^+^ T_reg_ cells, and unconventional T cell populations (Fig. S2). The relatively low levels of transcriptional activation and paucity of enriched meta-clones we observed in CD4^+^ T_CM_ cells aligned well with this observation (Fig. 1 and 2). The HLA^hi^ CD4^+^ T cell cluster had an identical “background” phenotype. Encouragingly, none of the other effector CD4^+^ or CD8^+^ T cell clusters mapped to background AIM^+^ T cells, supporting their identification as true antigen-responsive cells.

Identification of unconventional T cells (MAIT cells and γδT cells) by AIM-seq appears to be driven by two processes. Firstly, unconventional T cells were contributors to background AIM^+^ populations, suggesting even unstimulated unconventional T cells may be captured using AIM markers. Secondly, unconventional T cells, particularly MAIT cells and Vδ2^+^ γδT cells, are cytokine responsive (40, 41). Feed-forward signaling over 24 hours, the length of our assay, can drive activation of these cells in a cytokine-dependent manner (Amini et al, co-submission). Consistent with this, there was clear evidence of cytokine signaling in these cells. In response to BNT162b2 immunization, activation of unconventional cells by this feed-forward mechanism is associated with vaccine reactogenicity (Amini et al, co-submission). Thus, including these cells in future analyses may reveal intriguing biology.

The functional heterogeneity of AIM^+^ CD4^+^ T cells was striking; AIM^+^ CD4^+^ T cells were not simply IFN-γ-producing T_H_1 cells. With the exception of TNF, cells within each cluster were not necessarily producing cytokines associated with the cluster phenotype (e.g., IFN-γ and T_H_1). Whether this reflects a technical issue (e.g., suboptimal timepoint) or a biologic trait remains to be determined. Regardless, identification of IL-4/IL-13-producing CD4^+^ T_H_2 cells is consistent with prior ICS-based analysis (4, 16). IL-17 production was also observed as a hybrid T_H_1/T_H_17 population. This Th1/Th17 (CD4^+^CXCR3^+^CCR6^+^ (42)) population was also enriched in response to spike peptide in flow cytometric phenotyping experiments. It is unclear if this population reflects conversion of T_H_17 into T_H_1 cells over time after vaccination, or a true hybrid phenotype. Our TCR analysis revealed considerable clonal overlap between CD4^+^ T cell clusters and evidence of interconversion over subsequent vaccine doses. Interconversion of T cell polarization states is an increasingly well-understood phenomenon (43), but the underlying cause for such functional heterogeneity and why cells would change polarization states from one vaccine dose to the next remains to be determined. Further work is required to understand the impact these diverse functional populations have on vaccine immunogenicity and efficacy.

Included within the functionally heterogenous AIM^+^ CD4^+^ T cells was a T_reg_ population. The exact nature of these AIM^+^ T_reg_ cells is unclear; based on TCR analysis of our data and reanalysis of a published dataset (32), they have negligible clonal sharing with effector CD4^+^ populations and do not form antigen-enriched TCR meta-clones. Thus, they are unlikely to be spike-specific, but also do not appear oligoclonal or enriched to a given antigen based on TCR feature similarity. Their exact specificity remains to be determined. However, as they made up a larger proportion of the stimulated AIM^+^ cells compared with background, consistent with a previous report (20), they are unlikely to be a simple contaminant, although our time course analysis suggests spontaneous activation is a factor. Functionally, they appeared to not be producing inhibitory molecules but instead were a source of the T cell survival-promoting cytokine IL-7. Further work is required to understand how T_reg_ cells may modulate the immunogenicity of these vaccines, as T_reg_ cells have been shown to promote formation of T cell memory in other contexts (44).

With the initial description of T cells that cross-react between seasonal “common cold” coronaviruses (CCCoVs) and SARS-CoV-2, there was interest in the contribution of these cross-reactive T cells to immunity and vaccine immunogenicity (recently reviewed in (34)). In our study, only a small proportion of post-vaccine spike-reactive clones corresponded to pre-existing clones. This is consistent with these pre-existing clones making a minor contribution to the vaccine response (45). Intriguingly, all pre-existing clones that were detected in the subsequent post-vaccine immune response were found in individuals from the BNT group, with no involvement in ChAd vaccinated individuals. A recent study examining a larger cohort of vaccinees and specifically studying the S_816-830_ cross-reactive CD4^+^ T cell response also found no recruitment of pre-existing responses following ChAdOx1 priming, while recruitment was observed with BNT162b2 priming (9). In our study, the BNT groups also had greater recruitment of T cells induced by dose 1 into the recall response following dose 2. Thus, while pre-existing cross-reactive immunity makes only a minor contribution to the overall vaccine T cell response, there appears to be a fundamental difference in how these two vaccines engage cellular immunity, and in particular recall of memory T cell populations.

Previous studies have reported increased frequencies of IFN-γ^+^ T cells after ChAdOx1 nCoV-19 vaccination compared with BNT162b2 (7–9), particularly after the first dose. Consistent with this, we observed that the predominant IFN-γ^+^ CD4^+^ T_H_1/T_FH_ cluster was more abundant following ChAdOx1 vaccination, while the numerically more abundant CD4^+^ T_H_1/T_H_17 trended towards an increased frequency in BNT162b2 vaccinees. Furthermore, the IFN-γ^+^ CD8^+^ T cell cluster also had elevated *IFNG* transcripts in the ChAd group relative to BNT. Thus, the AIM assay indirectly recapitulates protein-level data from ELISpot and ICS. The overall frequency of AIM^+^ T cells was not different based on vaccine platform, demonstrating the capacity of different T cell assays to detect different populations (and thus frequencies) of functional T cells. In many other regards, the two vaccines induced markedly similar T cell responses. However, when interval between doses was considered, a more complex picture emerged.

Regardless of vaccine platform, extending the interval between first and second dose of vaccine increases post-boost antibody titers, but is associated with a modest expansion of IFN-γ^+^ T cell frequencies (5, 15). Thus, we had expected to see only minor differences in T cell phenotype after dose 2 based on interval. However, interval had the largest impact on T cell phenotype and the effect varied by vaccine platform. We (Amini et al, co-submission) and others (46–48) have noted increased innate inflammatory signaling after mRNA vaccine dose 2, which appears strongly driven by reactivation of memory T cell responses (Amini et al, co-submission) (49). Increasing the interval between doses dampens this response (Amini et al, co-submission). Thus, we hypothesize that after short BNT162b2 vaccination the elevated inflammatory environment induced by the second dose leads to a recall T cell response with increased IFN-γ and TNF signaling. Consistent with this, there was an elevated inflammatory response after dose 2 compared with dose 1 in BNT vaccinees, ruling out time since vaccination as the explanation for the phenotype. By contrast, inflammation induced by Ad vectors is driven primarily by engagement of innate pDCs and myeloid cells (50, 51) and thus would vary less between priming and boosting.

In contrast to the marked differences in T cell phenotype after dose 2 of short interval ChAd vs BNT, T cell responses were more similar between the two vaccines on the long interval regimen. For both vaccines, the post-boost T cell response appeared less effector-like with reduced evidence of inflammatory gene signaling. The relative contribution of enhanced central memory conversion due to the longer time before boost versus dampened innate immune signaling remains to be determined. For both vaccines, the longer interval between doses was associated with greater expansion of recalled clones, consistent with underlying differences in the T cell populations responding after dose 2. The exact functional impact of this altered T cell phenotype on immunity remains to be determined. However, as elevated inflammatory signaling drives CD4^+^ T cells away from a T_FH_ polarization state (52), it is possible that this lower inflammatory T cell response after boost may positively contribute to the enhanced antibody responses observed with the extending dosing interval. Conversely, the elevated interferon signaling in activated T cells after a short boosting interval may contribute to the high efficacy against severe COVID-19 seen with this regimen, despite markedly reduced antibody titers compared with long-interval boosting (53).

Inflammatory signaling in T cells is associated with increased effector polarization at the expense of long-lived proliferative memory differentiation (54). Given the elevated inflammatory phenotype of short-interval mRNA vaccination, we hypothesize T cells induced by this regimen would have reduced proliferative potential. Consistent with this, IFN-γ T cell responses generated by two doses of mRNA vaccination given in a long-interval increased in magnitude following an additional (third) dose, whereas those generated by a short-interval mRNA regimen did not (55). In our dataset, we observed an expansion of recalled clones after two doses of vaccine given in a long-interval vaccine regimen, but no expansion of recalled clones after a short-interval regimen, and this corresponded to increased expression of stem-associated genes in several CD4^+^ T cell clusters. Thus, the dosing interval for a primary vaccination regimen has a long-term impact on the anamnestic potential of vaccine induced T cells, which may lead to the increased long-term protection against COVID-19 observed with the extended-dosing interval (12).

In sum, we have demonstrated the utility of combining an AIM assay with scRNA-seq and scTCR-seq (AIM-seq) to characterize the phenotype, function and clonality of antigen-responsive T cells, in a manner spanning all epitopes within SARS-CoV-2 spike. While a few studies have used this approach in more limited ways, this study fully highlights the use of this approach for studying human T cell responses, particularly CD4^+^ T cell responses, in multiple contexts. Applying this assay, we defined key similarities and differences in the T cell response induced ChAdOx1 nCoV-19 and BNT162b2 vaccination and mild SARS-CoV-2 infection. Most strikingly, dosing interval between doses 1 and 2 had a marked, and platform-specific, impact on the resultant vaccine-induced T cell response. Combining these data with our co-submitted manuscript (Amini et al, co-submission), we have built a model where elevated inflammatory T responses in the short 3-4-wk BNT162b2 regimen is associated with enhanced innate responses at the time of boosting. These have practical considerations for optimizing dosing regimens of mRNA technology dependent on the context of its use – e.g., for use in infectious diseases or cancer, compared with use in autoimmunity or for gene therapy.

### Limitations

The primary limitation of the current study is the sample size per experimental group. While the overall dataset includes 60 samples for the AIM-seq assay (30 individuals and two timepoints) the inclusion of five different experimental groups limits the power of comparisons when performed at the level of individuals. Furthermore, the relatively limited number of cells captured per individual may lead to overestimation of the number of singlet clones identified. As we sorted AIM^+^ T cells and do not also have scRNA-seq data on whole peripheral blood samples, we are limited to analysis of cell-cell interactions only between T cells and with other important antigen-presenting cells. In addition, the use of 15mer peptide pools may bias towards the detection of CD4^+^ T cells.

## Materials and Methods

### Study design

We investigated the phenotype, function, and clonality of SARS-CoV-2 spike-specific T cells in humans after COVID-19 vaccination or SARS-CoV-2 infection. Specifically, we compared across different vaccine platforms (mRNA-LNP vs. adenoviral vector) and dosing intervals (3–4 weeks vs. 8–12 weeks). Peripheral blood was collected from healthy donors without previous symptomatic COVID-19 before and after two doses of BNT162b2 or ChAdOx1 nCoV-19 vaccination, and at analogous timepoints to vaccine dose one after mild COVID-19 in unvaccinated individuals. Single-cell CITE-and TCR-sequencing was performed on sorted AIM^+^ T cells after *ex vivo* stimulation of peripheral blood mononuclear cells with SARS-CoV-2 spike peptides. In addition, we assessed the contribution of pre-existing SARS-CoV-2-responsive T cells to the vaccine response by performing single-cell RNA- and TCR-sequencing on T cells that proliferated in response to SARS-CoV-2 spike peptides in blood sampled prior to vaccination. The study was not randomized, and investigators were aware of study groups.

### Ethics statement

Informed written consent was received from all participants. All work was performed in accordance with relevant ethical regulations and in compliance with the principles of the Declaration of Helsinki (2008). Protective Immunity from T Cells in Healthcare workers (PITC) ethical approval: Oxford GI Biobank Study Ethics Committee (REC Ref: 16/YH/0247, Yorkshire & The Humber Sheffield REC, approved on 29 July 2016, amended on 8 June 2020)). COV001 trial registration: NCT04324606; ISRCTN 15281137.

### Cohort and PBMC collection

Peripheral blood mononuclear cells (PBMCs) were collected from participants in COV001 Phase 1/2 clinical trial (56) and the PITCH (15) studies who received two doses of either BNT162b2 or ChAdOx1 nCoV-1 vaccines at long (8-12 weeks) or short (3-4 weeks) dosing intervals, or had mild COVID-19 infection (pre-Alpha COVID-19 strain). Samples from the short ChAd group were from participants in COV001, while samples from all other groups were from the PITCH study.

Participants were sampled before first vaccine (“T0”), and immediately before (“T1”) and four weeks after second (“T2”) COVID-19 vaccine. Patients with mild COVID-19 were diagnosed by SARS-CoV-2 PCR positivity and were sampled at timepoints selected to match the short interval vaccine dosing, such that T1-Short was 3-4 weeks and T1-Long was 8 weeks after initial PCR positivity. The age and sex of participants was similar across each experimental group (Table S1). PBMCs were processed and stored using similar protocols as in (15, 56).

### Stimulation, flow cytometric staining and sorting of activation induced marker T cells

One vial of cryopreserved PBMCs per patient per timepoint was thawed and washed in R10 (RPMI-1640 + 10% FBS + 1% Penicillin/Streptomycin) + Benzonase (500 U) on the day of use. Both timepoints of a single patient from each experimental group were included on a given day to minimize batch effects across sequencing runs. PBMCs from each vial were split in R10 across up to 8 wells of a U-bottom 96-well plate at 1×10^6^ PBMCs per well. Pools of overlapping peptides (15mers with 11 amino acid overlap) which covered the entire SARS-CoV-2 spike S1 and S2 protein regions were added to each well at a final concentration of 1 μg/ml and plates were incubated for 24 hours at 37 °C. One well of unstimulated cells per donor was included for use as a gating control for flow cytometric cell sorting. After incubation, plates were centrifuged at 1800 rpm for 2 min, washed in FACS buffer (PBS + 0.05% bovine serum albumin + 1 mM EDTA), and centrifuged again at 1800 rpm for 2 min prior to staining with the following antibodies: FITC-CD19 (HIB19), FITC-CD14 (M5E2), PE-4-1BB (CD137; 4B4-1), PE-OX-40 (CD134; Ber-ACT35), AF700-CD8α (SK1), BV421-CD69 (FN50), BV650-CD4 (OKT4), BV785-CD3 (OKT3), TotalSeq-C0032-CD154 (CD40L; 24-31), TotalSeq-C0155-CD107a (H4A3), TotalSeq-C0088-PD-1 (EH12.2H7), TotalSeq-C0171-ICOS (C398.4A), TotalSeq-C0063-CD45RA (HI100), TotalSeq-C0148-CCR7 (G043H7), TotalSeq-C0080-CD8 (RPA-T8), TotalSeq-C0072-CD4 (RPA-T4) and one of TotalSeq-C anti-human Hashtag antibodies (1-10; LNH-94, 2M2) per donor and timepoint. All antibodies were from BioLegend. TotalSeq antibodies were prepared as per manufacturer instructions and staining was performed in a final volume of 50 μl for 30 min at 4 °C. After staining, cells were washed three times with FACS buffer, resuspended in 100 μl of FACS buffer, and transferred to a 1.5 ml RNase-free Microfuge tube. SYTOX Green was pre-diluted 1:60 in PBS + 0.04% BSA, then diluted 1:100 in each sample. Samples were stored at 4 °C until sorting.

Sorting was performed on a BD FACSAria III (BD Biosciences) using an 85-micron nozzle. All AIM^+^ cells (Fig. 1A) from all individual samples were sorted into a single collection tube (RPMI-1640 + 1% NEAA + 1% Na Pyruvate + 2.5% HEPES + 10% FBS), washed twice in collection media and resuspended at 13,700 cells per 38.7 μl of resuspension buffer in preparation for the 10x Genomics Chromium Next GEM Single Cell 5’ v2 (Dual Index) workflow.

For flow cytometry-based characterization of AIM^+^ T cells, stimulations were performed as described above. Stimulation with an MHC-II-optimized immunodominant peptide pool for cytomegalovirus, Epstein Barr virus, influenza and tetanus toxoid (CEFT-II) was included as an additional stimulation. Samples were collected after 12 or 24 hours of stimulation. After incubation, plates were centrifuged at 1800 rpm for 2 min, washed in FACS buffer (PBS + 0.05% bovine serum albumin + 1 mM EDTA), and centrifuged again at 1800 rpm for 2 min prior to staining. Chemokine receptor staining was performed for 30 min at 37 °C with: PerCP-Cy5.5-CCR6 (G034E3), BV786-CCR7 (G045H7), BUV395-CXCR3 (1C6), PE-Cy7-CCR4 (L291H4).

Cells were washed once, and surface stained with: FITC-Vδ1-TCR (REAL277), BV421-CD69 (FN50), BV510-CD45RA (HI100), BV605-Vα7.2-TCR (3C10), BV711-Vδ2-TCR (B6), BUV563-CD3 (UCHT1), BUV737-CD127 (HIL-7R-M21), APC-CD161 (191B8), AF700-CD8α (SK1), Near-IR vital exclusion dye, APC-Cy7-CD19 (HIB19), APC-Cy7-CD14 (M5E2), PE-OX-40 (ACT35), PE-4-1BB (4B4-1), and PE-Cy5-CD4

(OKT4). Staining was performed in a final volume of 50 μl for 30 min at 4 °C. Cells were washed once and resuspended in 200 μl True-Nuclear 1× Fix Concentrate (BioLegend) and incubated light-protected for 60 min at room temperature. Cells were washed twice with True-Nuclear 1× Perm Buffer (BioLegend). Transcription factor staining for FoxP3 (PE-CF594, clone 206D) was performed in a final volume of 50 μl for 30 min at 4 °C. Samples were washed twice with True-Nuclear 1× Perm Buffer, resuspended in FACS buffer and stored at 4 °C until data acquisition on a BD LSRFortessa X-20 flow cytometer. Flow cytometry data was analyzed in FlowJo v10.

### CellTrace Violet assay to detect pre-existing SARS-CoV-2 specific clones

Given its high sensitivity to detect pre-existing SARS-CoV-2 spike cross-reactive T cells, we used a CellTrace Violet (CTV) dilution assay (35). One vial of cryopreserved PBMCs per vaccinee at the pre-vaccination (baseline; “T0”) timepoint was thawed and washed in R10 (RPMI-1640 + 10% FBS + 1% Penicillin/Streptomycin) + Benzonase (500 U) on the day of use. 5.25 × 10^6^ cells per donor were collected and labeled with CTV, as described (35). Briefly, cells were pelleted and washed twice with sterile PBS. After washing, cells were resuspended in 1 ml of PBS and 0.5 μl of CTV was added (1:2000 dilution). Cells were stained for 10 minutes at room temperature and the reaction was quenched using 4 ml of ice-cold FBS. Media was removed and cells were resuspended in 5 ml of RhuAB10 media (RPMI-1640 + 10% human AB serum + 1% Penicillin/Streptomycin). Cells were allowed to rest for 5 min to allow excess CTV to leach away, counted, and resuspended in fresh RhuAB10 media at 2.5 × 10^6^ cells per ml.

Cells (2.5 × 10^5^) were added to wells of a 96-well U-bottom plate. Unstimulated control (containing DMSO) and positive control (PHA at 2 μg/ml) wells were included. The remainder of the cells were added in sequential wells so that all cells were used and stimulated with S1 + S2 peptide pools (each at 1 μg/ml) in a final volume of 200 μl. Cells were placed in a 37 °C, 5% CO_2_ incubator for 7 days. On day 4, the plate was centrifuged (1800 rpm for 3 min) and 100 μl of media was removed and replaced with fresh, pre-warmed RhuAB10 media.

On day 7, the plate was centrifuged (1800 rpm for 2 min), and all media was removed. Cells were washed in FACS buffer and wells were merged (3 into 1) to reduce the number of wells to stain. Staining was performed for 30 min at 4 °C using a cocktail of: FITC-CD19 (HIB19), FITC-CD14 (M5E2), AF700-CD8α (SK1), BV650-CD4 (OKT4), BV785-CD3 (OKT3), and one of TotalSeq-C anti-human Hashtag antibodies (1-10; LNH-94, 2M2) per donor. TotalSeq-C antibodies were prepared as described above. After staining, cells were washed three times with FACS buffer, resuspended in 100 μl of FACS buffer, and transferred to a 1.5 ml RNase-free Microfuge tube.

SYTOX Green was pre-diluted 1:60 in PBS + 0.04% BSA, then diluted 1:100 in each sample. Samples were stored at 4 °C until sorting.

Sorting was performed as per AIM^+^, using the gating highlighted in Fig. 3A. Flow cytometry data was analyzed in FlowJo v10.

### Single-cell RNA-sequencing library preparation and sequencing

Samples were loaded onto a 10x Chromium Controller at an average of 23,000 cells per channel (28 channels total). Gene expression, cell surface protein (ADT) and TCR sequencing libraries were prepared as per manufacturer guidelines, and sequencing was performed on an Illumina Novaseq 6000 as per the manufacturer’s instructions (Wellcome Sanger Institute, Cambridge, UK).

### Data analysis methods

#### Statistical comparisons

Where indicated in the figure legends, parametric and non-parametric statistical comparisons were performed after assessing the data for normality by direct visualization of the data distribution and a Shapiro-Wilk test when sample sizes permitted. Boxplots are median and interquartile range and whiskers are 1.5x interquartile range. Statistics used are listed in figure legends.

#### Read mapping, quality control, hashtag demultiplexing and clustering

FASTQ files were generated from BCL files using Illumina bcl2fastq. FASTQ files for all modalities were mapped to the GRCh38-2020-A reference genome and a custom ADT marker list using the Cell Ranger 7.0.0 multi pipeline for count, ADT and VDJ data. The filtered_contig_annotations.csv file was filtered to retain only high-confidence, full-length, productive contigs corresponding to TCRα or TCRβ chains.

Data analysis was primarily performed using the Seurat package (v4.3.0) (57) in R (v4.3.0). Quality control was performed as follows: low quality cells were removed (based on low UMI and gene count and high percent mitochondrial reads), doublets were flagged and removed using scDblFinder (58). Immunoglobulin and TCR gene segments, identified from the IMGT database (imgt.org), were removed from the gene expression object, except for the following genes: *IGHM, IGHG1, IGHG2, IGHG3, IGHG4, IGHD, IGHE, IGHA1, TRAV1-2, TRAV24, TRDV1, TRDV2, TRDV3*. Hashtags for each unique individual within a single experiment were demultiplexed using MULTIseqDemux (59) and then data from each experiment were merged into a single Seurat object. Gene counts were log-normalized (NormalizeData function), variable features were found (n=2000, FindVariableFeatures function) and features were scaled using default Seurat parameters (ScaleData function). The top variable features were used for nearest-neighbor and Louvain clustering (resolution 0.7) and to generate principal components, the top 30 of which were used to generate uniform manifold approximation and projections (UMAP). One individual was identified in the vaccine cohort that had potential previous asymptomatic COVID-19 infection. A sensitivity analysis was performed that indicated that this individual did not differ from other individuals in the vaccine group at either T1 or T2, as previously reported (60), therefore the potentially previously infected donor was retained for further analysis.

Following Louvain clustering and expert annotation of identified cell clusters, several developmentally distinct cell populations were found to co-cluster based on gene expression and were manually separated as follows: MAIT_2 were separated from the co-clustered VD2 and CD8_Tem populations based on co-expression of SLC4A10 and TRAV1-2; VD1_Eff were separated from the co-clustered CD8_IFNG population based on lack of expression of abTCR chains.

#### Baseline spike-responsive T cell data pre-processing

One individual was removed from the baseline spike-responsive T cell data analysis due to detection of potential previous SARS-CoV-2 infection. Single-cell RNA-sequencing data on CTV^lo^ baseline reactive T cells underwent QC as per above. Integration of data from different experiments (one donor per experimental group was included per experiment) was performed using Seurat IntegrateData with 3000 variable features selected. The integrated data slot was used for subsequent data visualization as appropriate. As in the AIM^+^ spike-responsive T cells, after Louvain clustering, we identified developmentally distinct populations which co-clustered. We therefore separated the VD2 population from CD8_IFNG and CD8_GZMA populations based on lack of expression of abTCR chains.

#### Differential abundance analysis

Differential abundance analysis was performed using the edgeR package (v3.42.2) (61), with the function glmQLFtest with flags robust=TRUE and abundance.trend=FALSE and dispersions estimated using estimateDisp with the flag trend=“none”.

#### Differential gene expression and geneset enrichment, overrepresentation and variation analysis

For comparisons between individuals in different groups, differentially expressed genes were defined using Wilcoxon rank sum test with p values corrected using Bonferroni correction based on the number of genes in the dataset which passed filtering with an average fold-change of >0.05 and minimum percent expression within each compared cluster of 25% (FindMarkers function). For within-individual comparisons (e.g. those across timepoints), differential expression of genes was assessed using the Seurat FindMarkers function with the test.use=“LR” flag, with individual as a latent variable.

For analysis of the interactions between vaccine, interval and timepoint, sex and age, mixed-effects linear models with estimated precision weights were assessed using the dream method of variancePartition (v1.3.0) (62), based on analysis performed in (63). Briefly, gene counts within a given individual * timepoint * cluster combination were aggregated using the edgeR function Seurat2PB, were filtered by expression with filterByExpr with min.count=5 and min.total.count=15, and normalized library sizes generated. voomWithDreamWeights was used to normalize data and estimate precision weights for dream analysis. Dream analysis was performed using the formula ~ 0 + group.time + Age + Sex + (1|ID) to estimate regression coefficients for each gene and empirical bayes shrinkage was applied using the eBayes function of variancePartition.

Genesets used for GSEA and overrepresentation analyses were obtained from Human MSigDB Hallmark or the Gene Ontology Biological Process database and filtered to include genesets related to cytokines, T helper and cytotoxic subsets, and antigen presentation pathways. A CD8^+^ T cell cytotoxicity associated geneset was obtained from (28). Stimulated MAIT cell genesets were obtained from (25) by taking the top 100 differentially expressed genes from sorted MAIT cells that were most differentiated from unstimulated cells after stimulation with a TCR (MR1/5-OP-RU), cytokine (IL-12 + IL-18), or TCR and cytokine stimulus. Gene lists were pre-ranked based on either average log fold change of differentially expressed genes or using the scaled coefficients (z.std) resulting from the contrasts applied following dream analysis. GSEA and overrepresentation analysis were performed using the fgsea function within the fgsea (v1.26.0) (64) or clusterProfiler (v4.8.1) packages (65). Published genesets related to CD8^+^ T cell cytotoxicity (28) were quantified using the Seurat AddModuleScore function with default parameters, or with geneset variation analysis (66) on aggregate, pseudo-bulked counts of the relevant cell type.

#### CellPhoneDB

To find putative cell-cell interactions, CellPhoneDB (v5, (67)) was applied to the entire pre-processed and filtered dataset, or subsets of cells including only BNT162b2 or ChAdOx1 nCoV-19 vaccinated individuals. The Seurat package FindAllMarkers function was used to define differentially expressed genes for each cluster within each dataset with flags only.pos=T, random.seed=1, logfc.threshold=0.25, return.thresh=0.05, min.pct=0.25. The function pdb_degs_analysis_method with threshold=0.1 was used to find interactions between celltypes. Downstream visualization and analysis was performed in R (v4.3.0).

#### TCR analysis

Paired abTCR chains were compiled for each cell using the scRepertoire package (v2.0.4), which loads filtered_contig_annotation files (output from 10x CellRanger multi) and combines them with the Seurat object for downstream analysis. Briefly, createHTOContigList was used with groups based on the called hashtag IDs and combineTCR with default settings. Clones were called based on paired CDR3 amino acid chain calling within each individual at each timepoint. Visualization of overlapping TCR clones was performed using the circlize package (v0.4.15) (68). Visualization of selected TCR amino acid sequences motifs was performed using the ggseqlogo package (v0.1). For analysis over time, clones were called as those with identical paired abTCR chains, and ‘recalled clones’ were defined as those present at more than one timepoint.

To identify the relationship between TCR physiochemical properties and gene expression, and identify clones enriched in our dataset compared with simulated ‘background’ TCR repertoires, we performed analysis using the CoNGA package (v0.1.2) (69) in Python (v3.9). The filtered and normalized Seurat object was exported from R into an anndata object using the DropletUtils package (v1.20.0) (70). Variable features were found and data was scaled using conga.preprocess.filter_and_scale, with parameters set to include all previously filtered cells. Cells were reduced to a single clone using reduce_to_single_cell_per_clone with default settings, which returns a single representative clone using a PCA-based approach to identify the single cell within a paired abTCR amino-acid clonotype with the least average gene expression difference to other cells within a clonotype. Within the CoNGA package, TCRdist was used to quantify physiochemical scores of the TCRs of each clonotype and find the distance in TCR-space between two independent TCR clonotypes. This metric was then used in kernel PCA to reduce the cell x cell TCRdist matrix. The 50 top TCRdist kernel PCs were retained for downstream clustering and dimensionality reduction, alongside the top 40 PCs of the variable gene expression. Neighborhoods of gene and TCRdist similarity were defined using conga.preprocess.calc_nbrs, with nbr_frac_for_nndists=0.01 and nbr_fracs=[0.01,0.1]. Comparison of the TCRdist and gene expression nearest neighbor graphs was then performed to compare gene expression and TCR distancing metrics with conga.correlations.run_graph_vs_graph. Significant CoNGA scores represent clonotypes that are present in neighborhoods with significant overlap in gene expression and TCR space.

To identify TCR meta-clonotypes that were likely to be antigen-enriched, we performed TCR clumping analysis using conga.tcr_clumping.assess_tcr_clumping with default settings. This analysis simulates a background VDJ repertoire representative of a simple model of VDJ recombination and tests whether individual TCR neighborhoods are overrepresented in the observed data compared with this null model of VDJ recombination. Meta-clonotypes with >10 participating clonotypes were selected for further analysis, and clustalOmega within the msa (v1.32.0) package was used to derive consensus sequences for ab CDR3 regions separately.

## Supplementary Material

Supplementary Material

## Data Availability

No custom software was generated for this manuscript. The software used for all analyses is listed in the relevant sections of the Methods and sufficient detail is given to reproduce analyses. All data required to reproduce figures are available in supplementary data file 1. Primary scripts are available on reasonable request. Raw data and the final, processed and annotated Seurat object from this study will be made available upon final publication (GEO XXXXX).

## One sentence summary

Antigen-specific T cell functionality is dependent on dose-interval between COVID-19 vaccines, and a longer dosing interval reduces T cell inflammation after two doses of mRNA-LNP vaccination.
