# Supplementary material for: The dominant impact of dosing interval on the quality of T cells induced by SARS-CoV-2 mRNA and adenoviral vaccines
Source: Sci Immunol. Author manuscript; Available in PMC 2026 Feb 23. (PMC7618773; doi:10.1126/sciimmunol.adu4610)
Supplement: Supplementary Material [file EMS212517-supplement-Supplementary_Material.pdf]

The dominant impact of dosing interval on the quality of T cells induced by SARS-CoV-2 mRNA and adenoviral vaccines

Sam M. Murray, Ali Amini, Helen Ferry, Lucy C. Garner, Maria Fransiska Pudjohartono, Barbara Kronsteiner, Sagida Bibi, Andrew J. Pollard, Eleanor Barnes, Teresa Lambe, Susanna Dunachie, Paul Klenerman, Nicholas M. Provine

Supplementary Materials

Supplementary figures 1-14

Supplementary tables 1 and 2

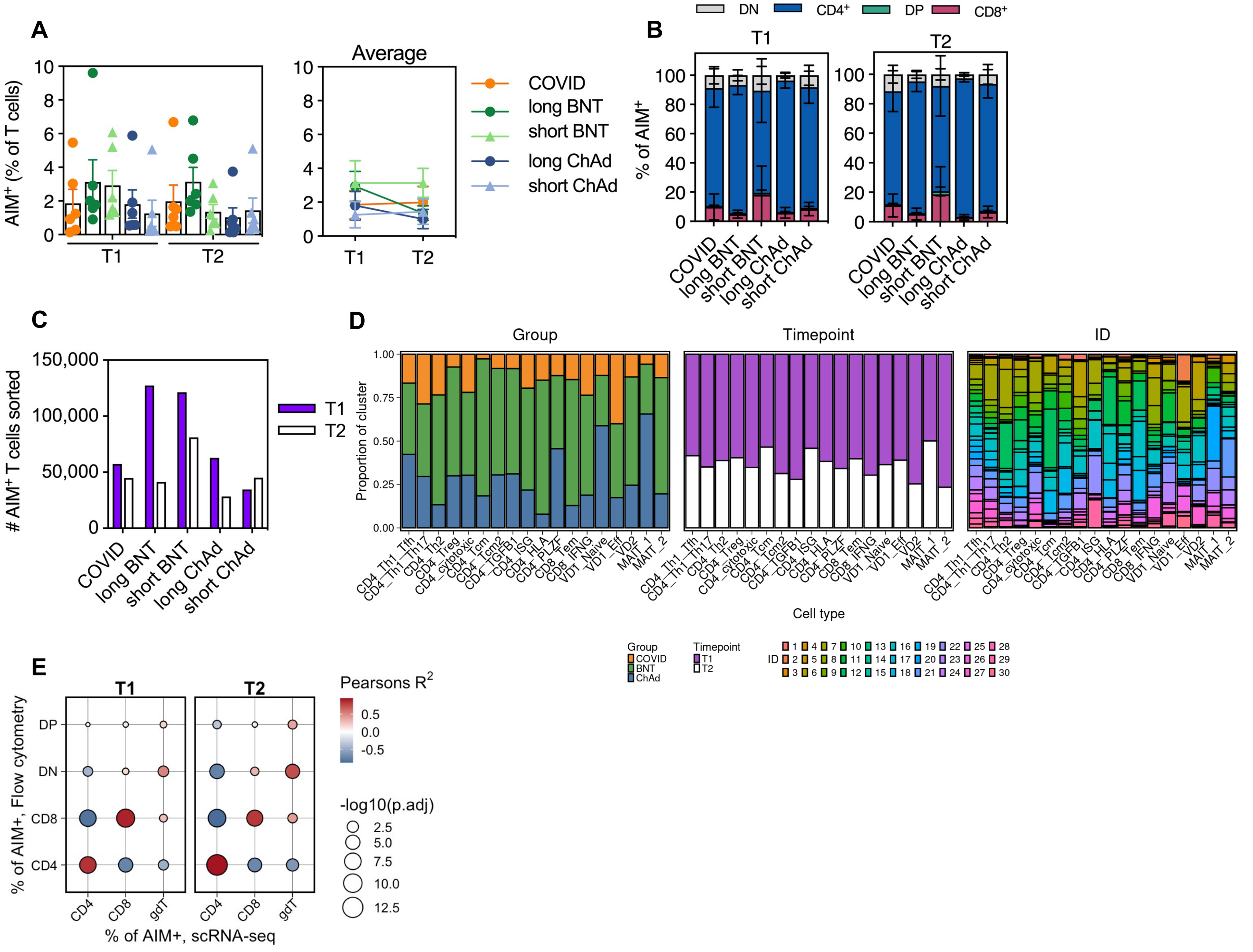

Fig. S1) A) Frequency of CD69⁺, 4-1BB⁺/OX-40⁺ (activation induced marker, AIM⁺) CD3⁺ T cells after stimulation with SARS-CoV-2 spike peptide. **B)** Frequency of CD4⁺, CD8⁺, CD4⁺CD8⁺ (Double positive, DP) and CD4⁻CD8⁻ (Double negative, DN) AIM⁺ CD3⁺ T cells. **C)** Total number of AIM⁺ T cells sorted for single-cell RNA sequencing. **D)** Proportion of study group, timepoint, and participant ID of AIM⁺ T cell clusters defined by single-cell RNA sequencing. **E)** Pearsons correlation of the frequencies of CD4⁺, CD8⁺, DP or DN within AIM⁺ measured by flow cytometry and frequencies of aggregate single-cell RNA sequencing clusters.

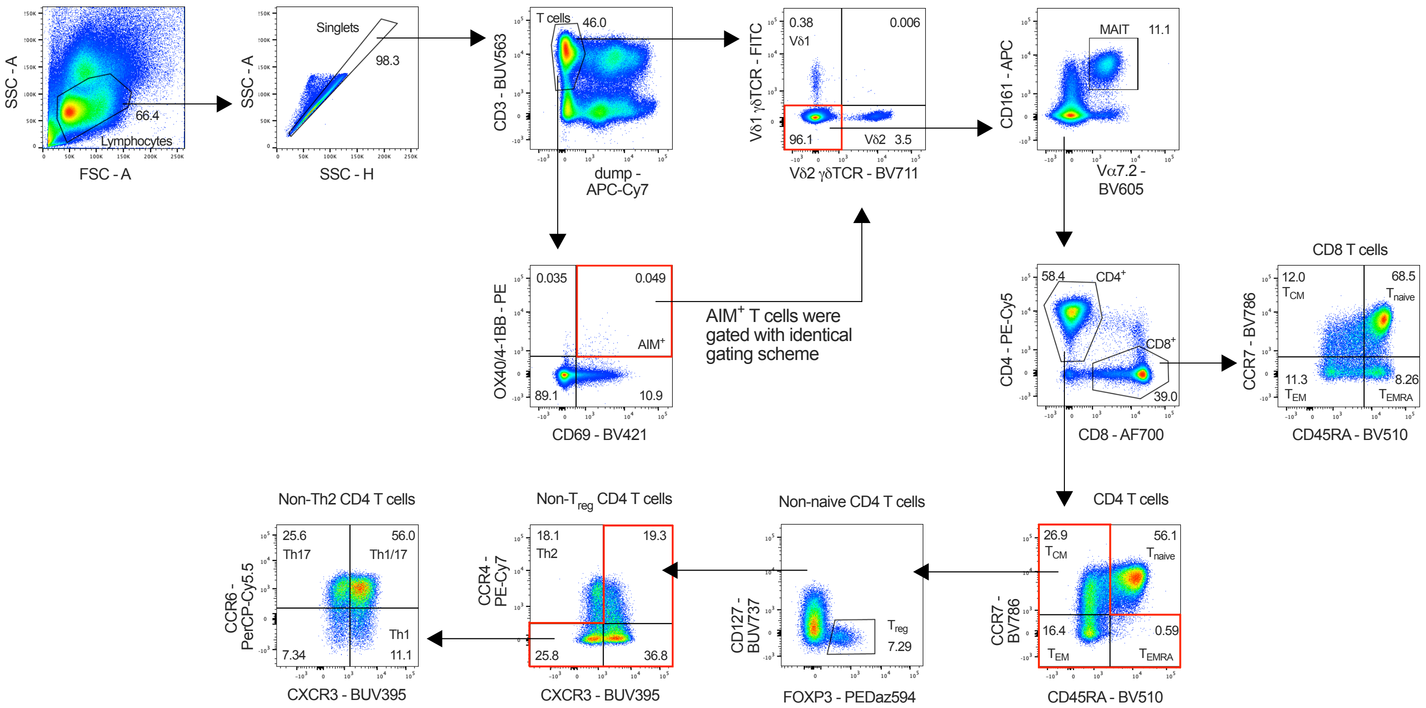

Fig. S2) Representative gating scheme for validation flow cytometry experiment. Performed in N=12 BNT vaccinees.

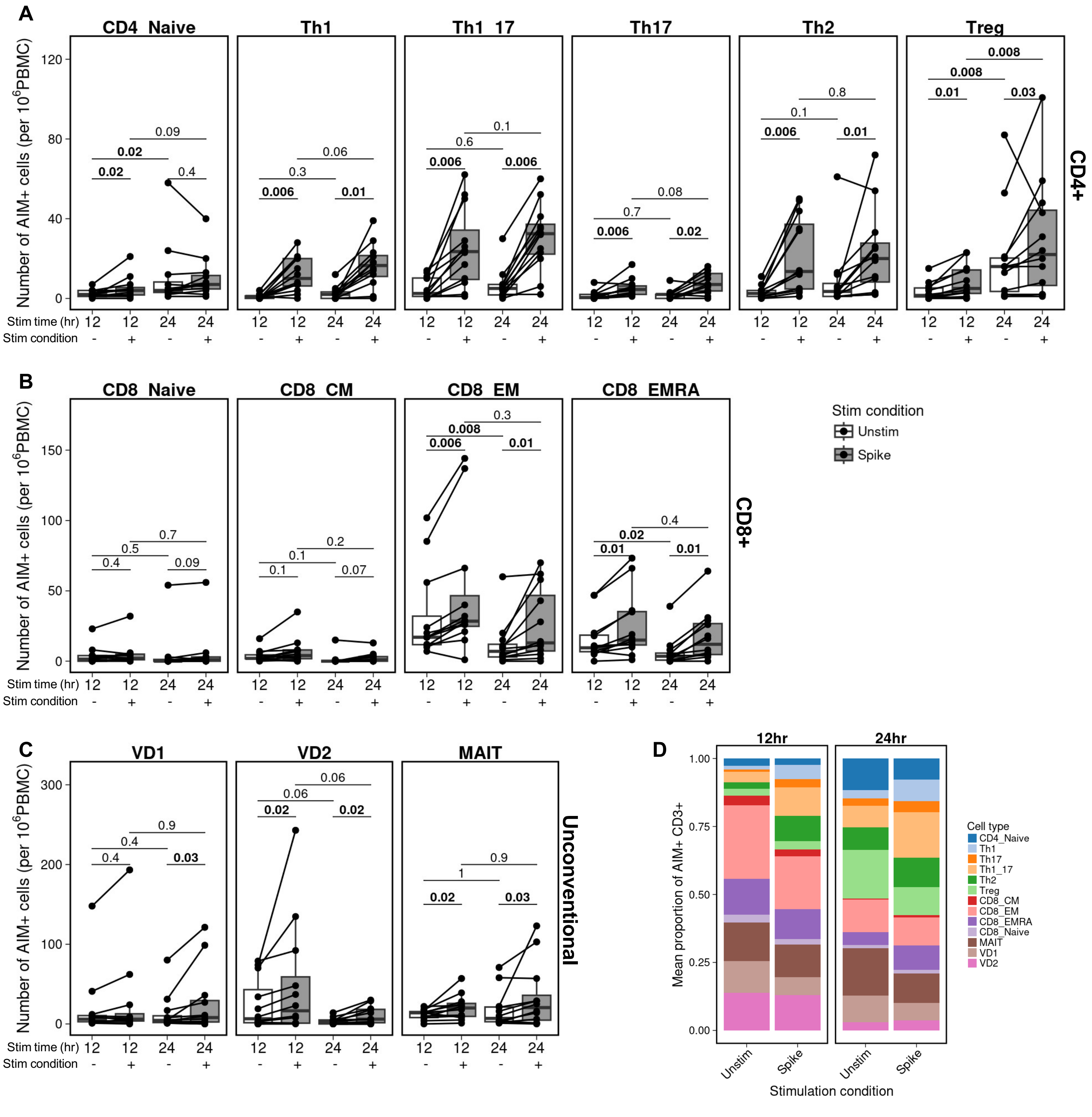

Fig. S3) Flow cytometric phenotyping of unstimulated and SARS-CoV-2 spike-responsive AIM$^+$ T cells. **A-C)** Number of activation induced marker positive (AIM)$^+$ (CD69$^+$ and OX-40$^+$ and/or 4-1BB$^+$) cells per 10^6 peripheral blood mononuclear cells (PBMCs) after 12- or 24-hour incubation with DMSO (Unstim, −) or SARS-CoV-2 spike peptide pools (Spike, +). N=12 pre-second vaccine (T1) from BNT vaccinees. Conventional CD4$^+$ (A), conventional CD8$^+$ (B) and unconventional (C) cell populations are presented. P values are Benjamini-Hochberg false-discovery rates (FDR) from paired or unpaired Mann-Whitney U tests. **D)** Mean proportion of T cell subsets amongst AIM$^+$ CD3$^+$ T cells.

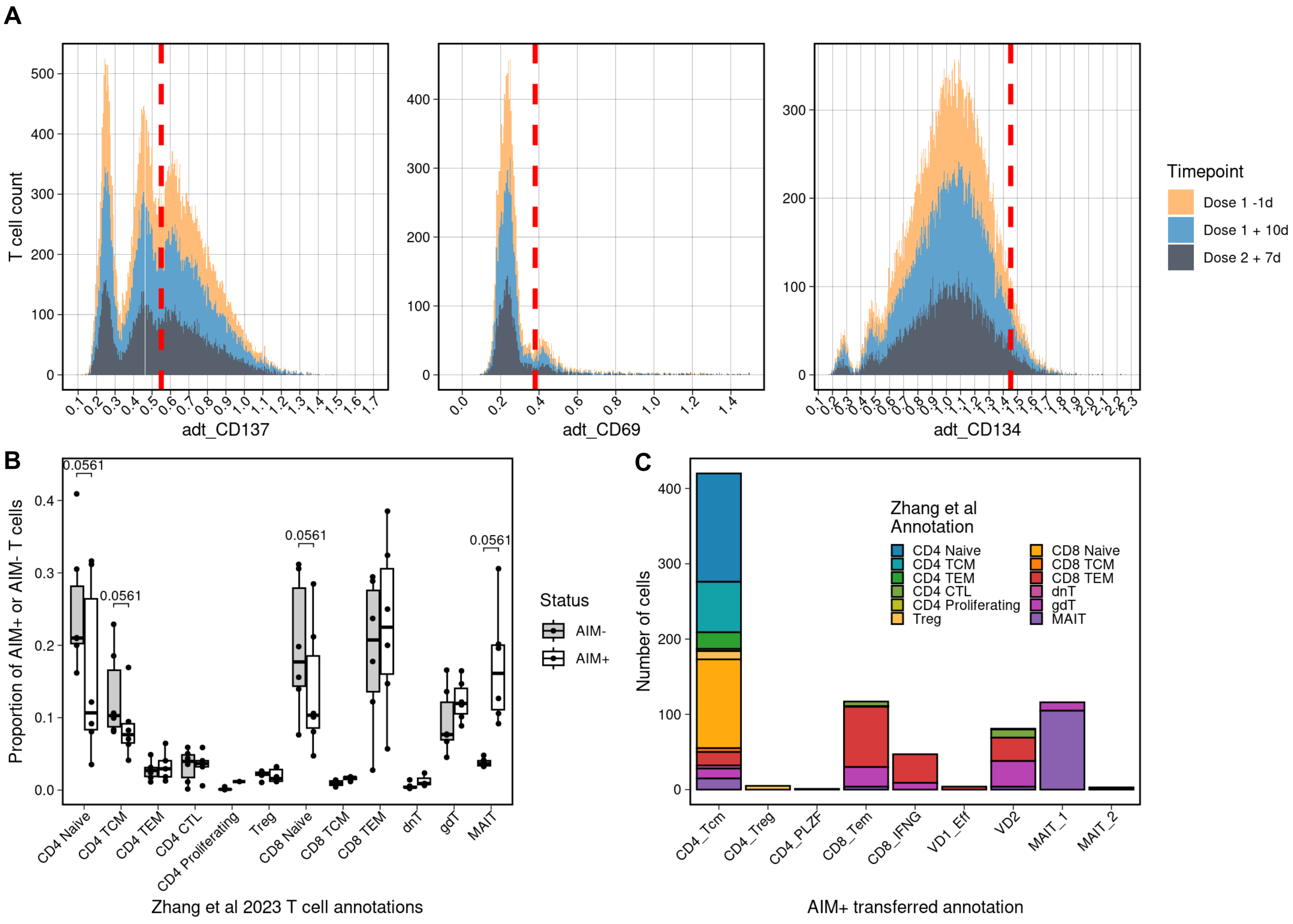

Fig. S4) Cellular indexing of transcriptomes and epitopes (CITE) sequencing reanalysis of RNA and surface protein expression on unstimulated T cells from dataset derived from Zhang *et al* 2023 (22). **A)** Normalized expression of activation induced marker surface expression on unstimulated T cells. For CD137 (4-1BB) and CD69, red lines represent manually defined expression cut-off. For CD134 (OX- 40), a clear positivity cut-off could not be defined, so a 95% expression level cut-off was used. **B)** Proportion of Zhang et al defined T cell subpopulations in AIM$^+$ (CD69$^+$, 4-1BB$^+$/OX-40$^+$, defined using thresholds in A) and AIM$^-$ T cells in the unstimulated T cells. **C)** Projection of annotations defined on spike-responsive AIM$^+$ T cells in the present study onto unstimulated AIM$^+$ T cells in Zhang *et al.* 2023 dataset.

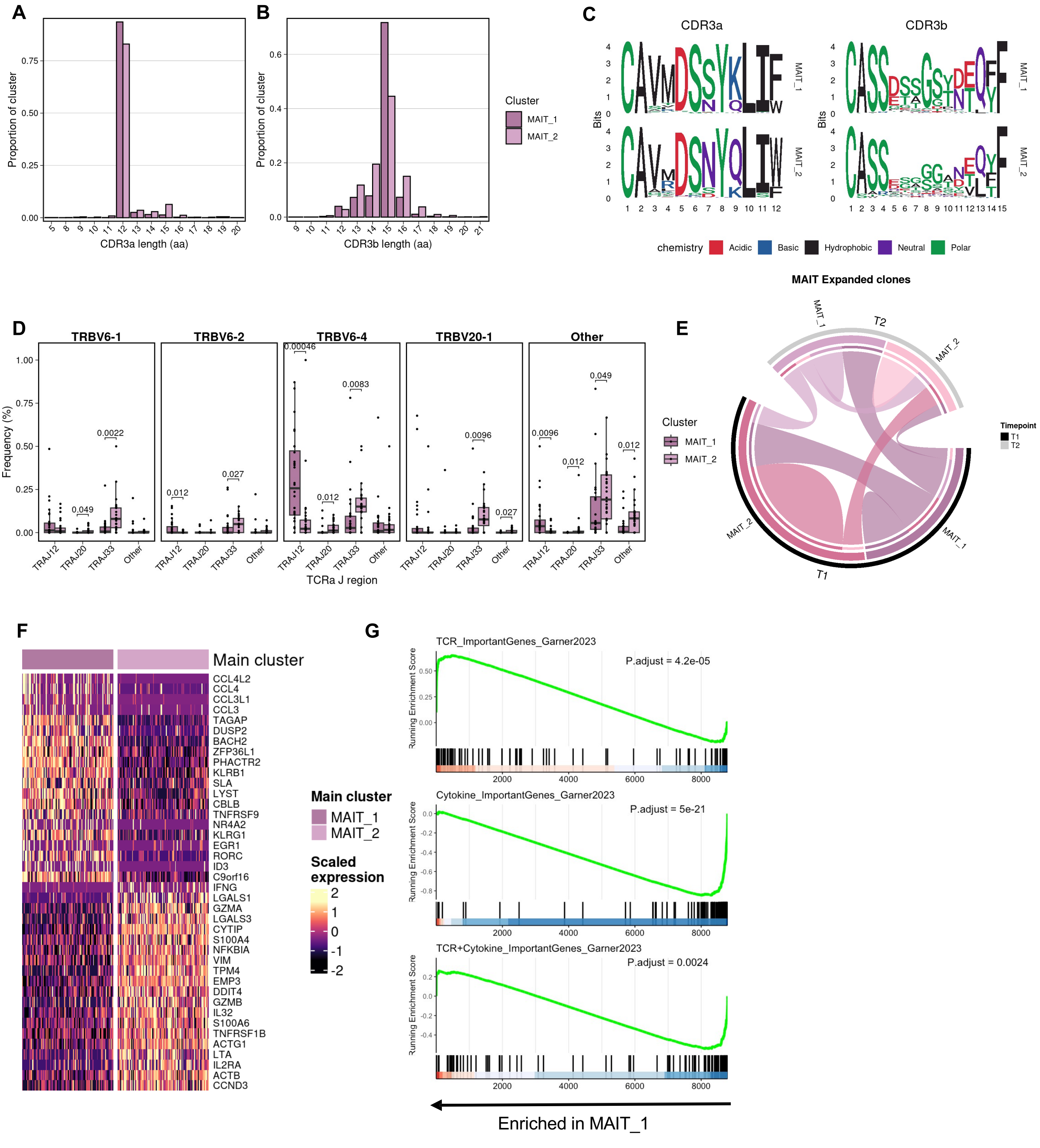

Fig. S5) CDR3 length of α **(A)** and β **(B)** TCR chains in MAIT_1 and MAIT_2 AIM⁺ T cell populations. **C)** CDR3 amino acid motifs of most common length α and β TCR chains from MAIT_1 and MAIT_2 AIM⁺ T cell populations. **D)** Frequency of TRAJ and TRBV gene usage combinations. **E)** Overlap of paired αβ CDR3 amino acid sequence clones between MAIT populations and across timepoints. **F)** Scaled RNA expression of the top 20 significant differentially expressed genes between MAIT subtypes. **G)** Geneset enrichment of genesets derived from sorted MAIT cells stimulated with TCR, cytokine (IL-12 and IL-18) or TCR and cytokine (IL-12 and IL-18) in (Garner *et al.* 2023 (25), **Methods**). Genes were ranked based on average log fold change between MAIT clusters.

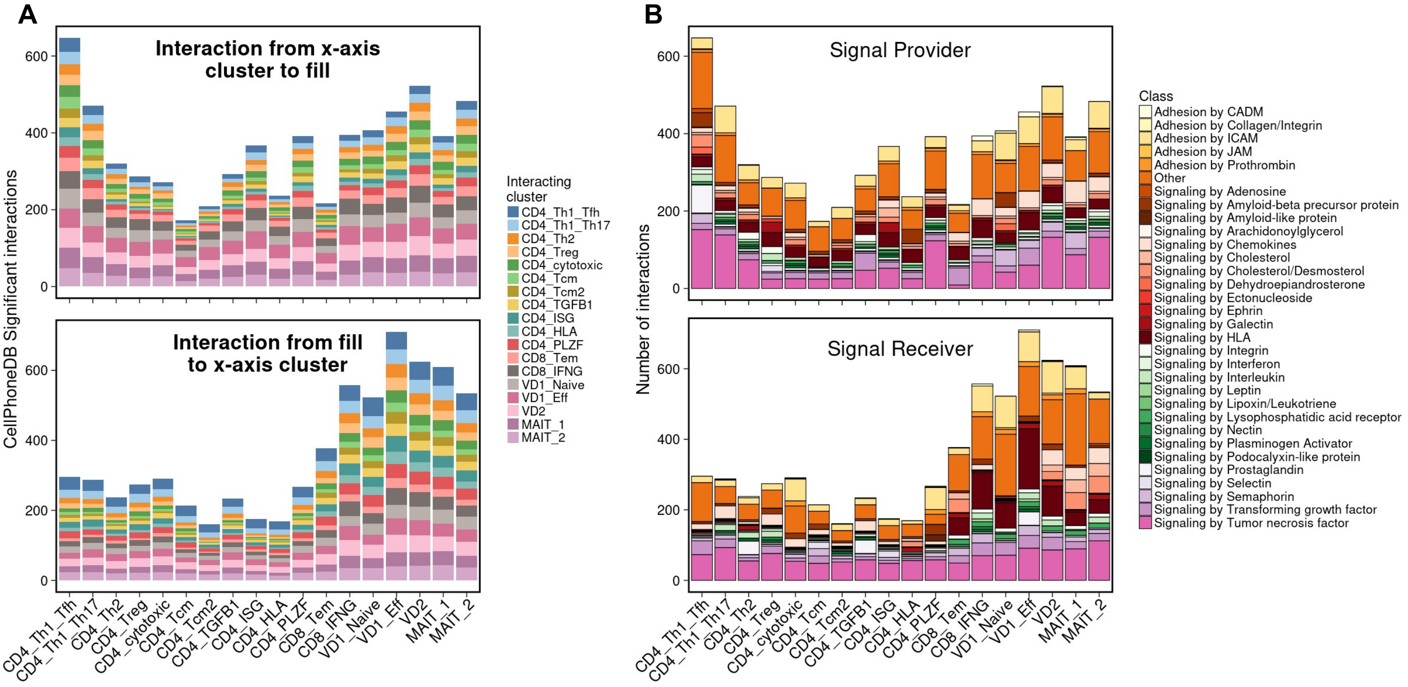

Fig. S6) Significant CellphoneDB derived interactions from (top) or to (bottom) each spike-responsive T cell cluster, separated by **A)** cell type interaction partner, or **B)** broad biological interaction class.

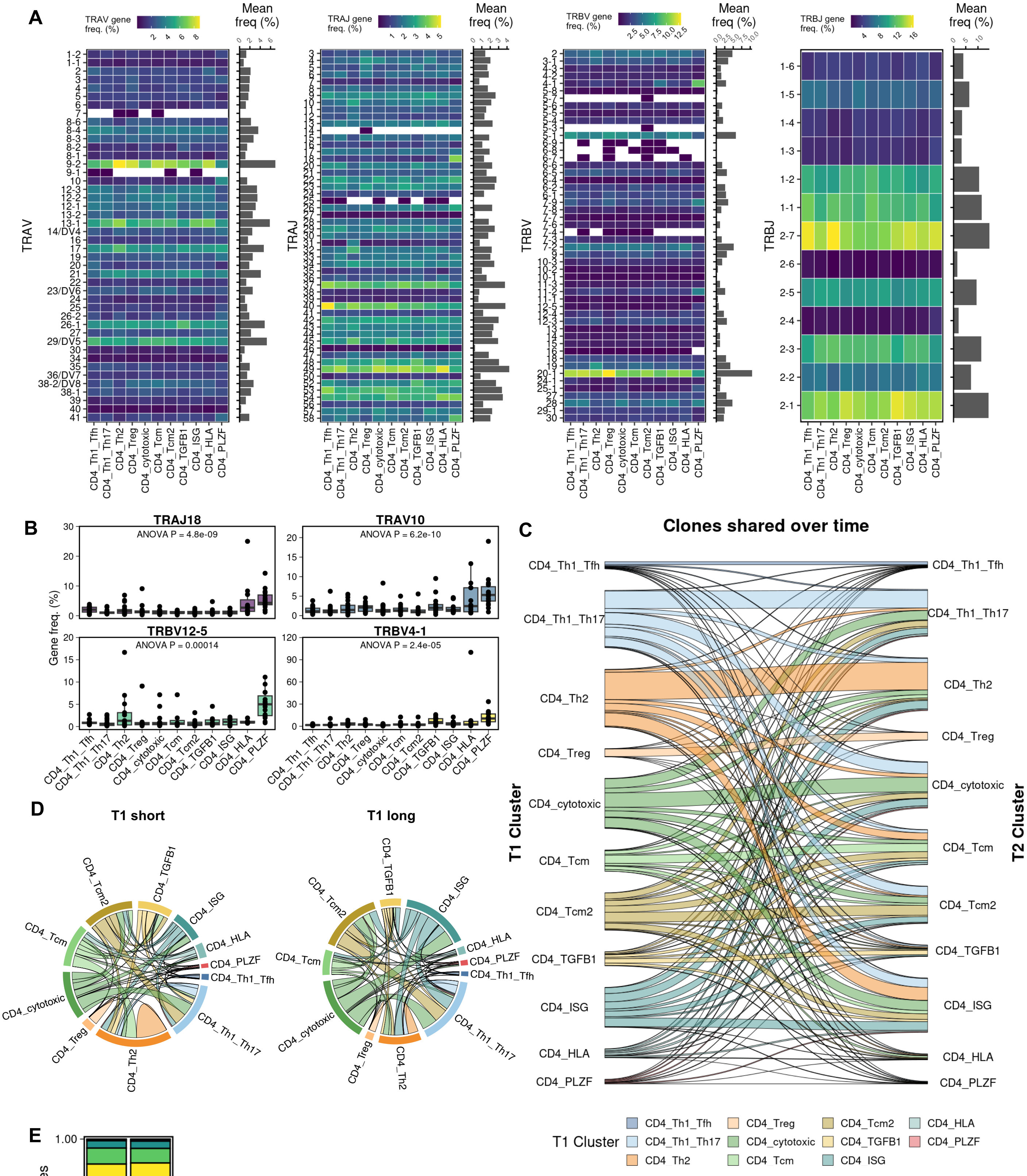

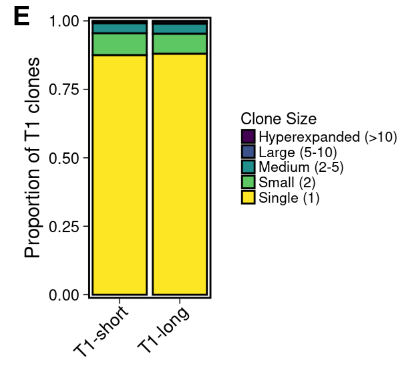

Fig. S7) A) TRAJ, TRAV, TRBJ and TRBV frequencies within spike-responsive CD4[+] cell populations. **B)** Gene usages with most significant differences in variation across cell clusters measured by ANOVA. **C)** Overlap of paired αβ CDR3 amino acid sequence clones between spike-responsive CD4[+] T cell populations across timepoints**. D)** Overlapping paired αβ CDR3 amino acid clones CD4 T cell populations at T1 from individuals in short (left panel) or long (right panel) interval dosing regimens. **E)** Proportion of expanded clones among T1 clones from short and long interval vaccinees.

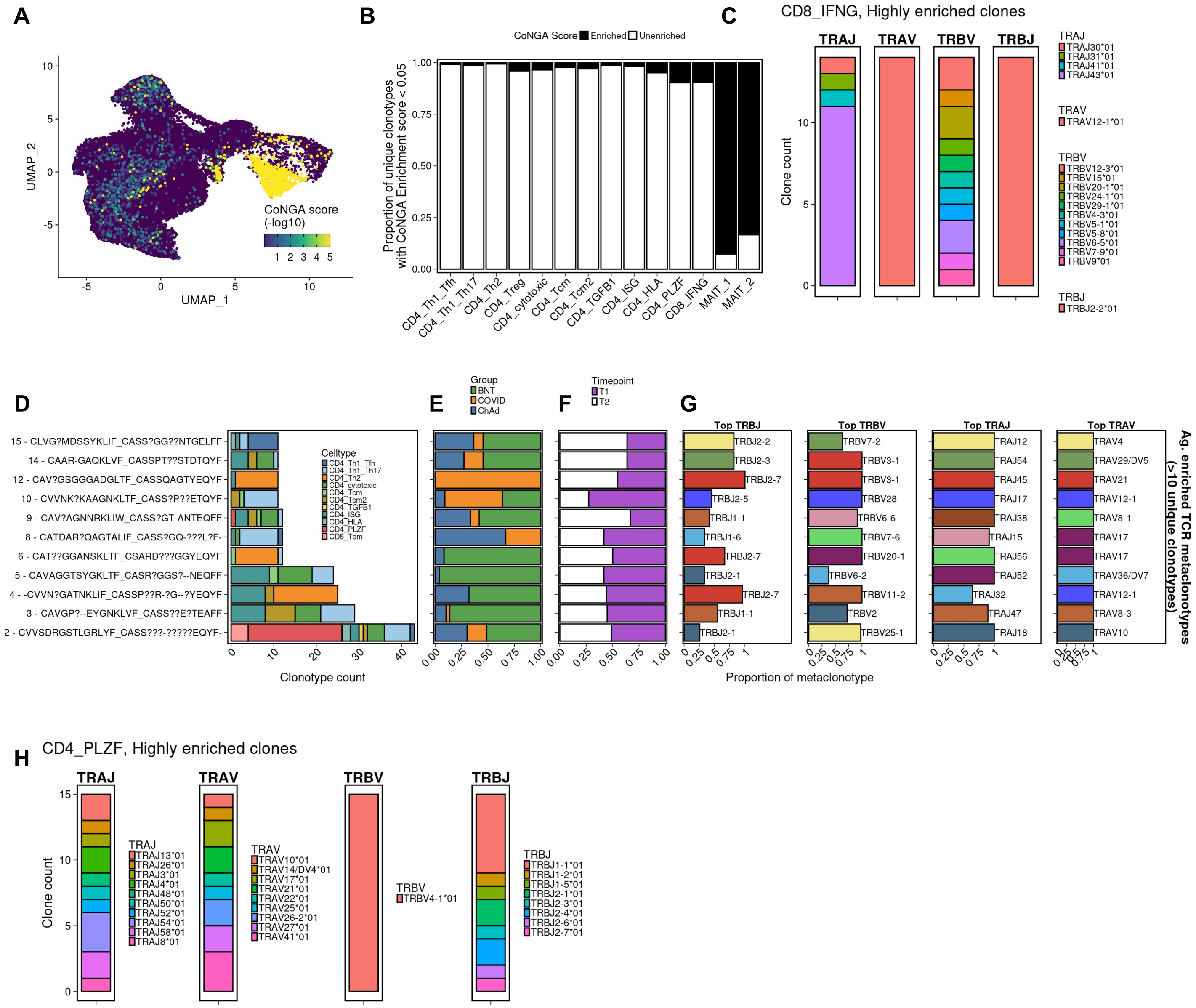

Fig. S8) CoNGA analysis of TCR and Gene expression. All data represent cells condensed into single unique clonotypes (n=61,375) **(Methods)** and Schattgen *et al.* 2022. **A)** Gene expression UMAP embedding colored by CoNGA score. Score capped at 10^{-5} to aid visualization **B)** Proportion of unique clonotypes with CoNGA enrichment score <0.05 (enriched). **C)** Analysis of gene usage from highest CoNGA enriched clones (CoNGA score <$1x10^{-10}$) within CD8_IFNG cluster. **D-G**) Antigen-enriched TCR meta-clonotypes with >10 participating unique clonotypes detected using TCR enrichment program within the CoNGA package, includes paired αβ CDR3 consensus sequences and cell type (D), vaccine (E), timepoint (F), and TCR gene usage proportions (G). **H**) Analysis of gene usage from highest CoNGA enriched clones (CoNGA score <$1x10^{-10}$) within CD4_PLZF cluster.

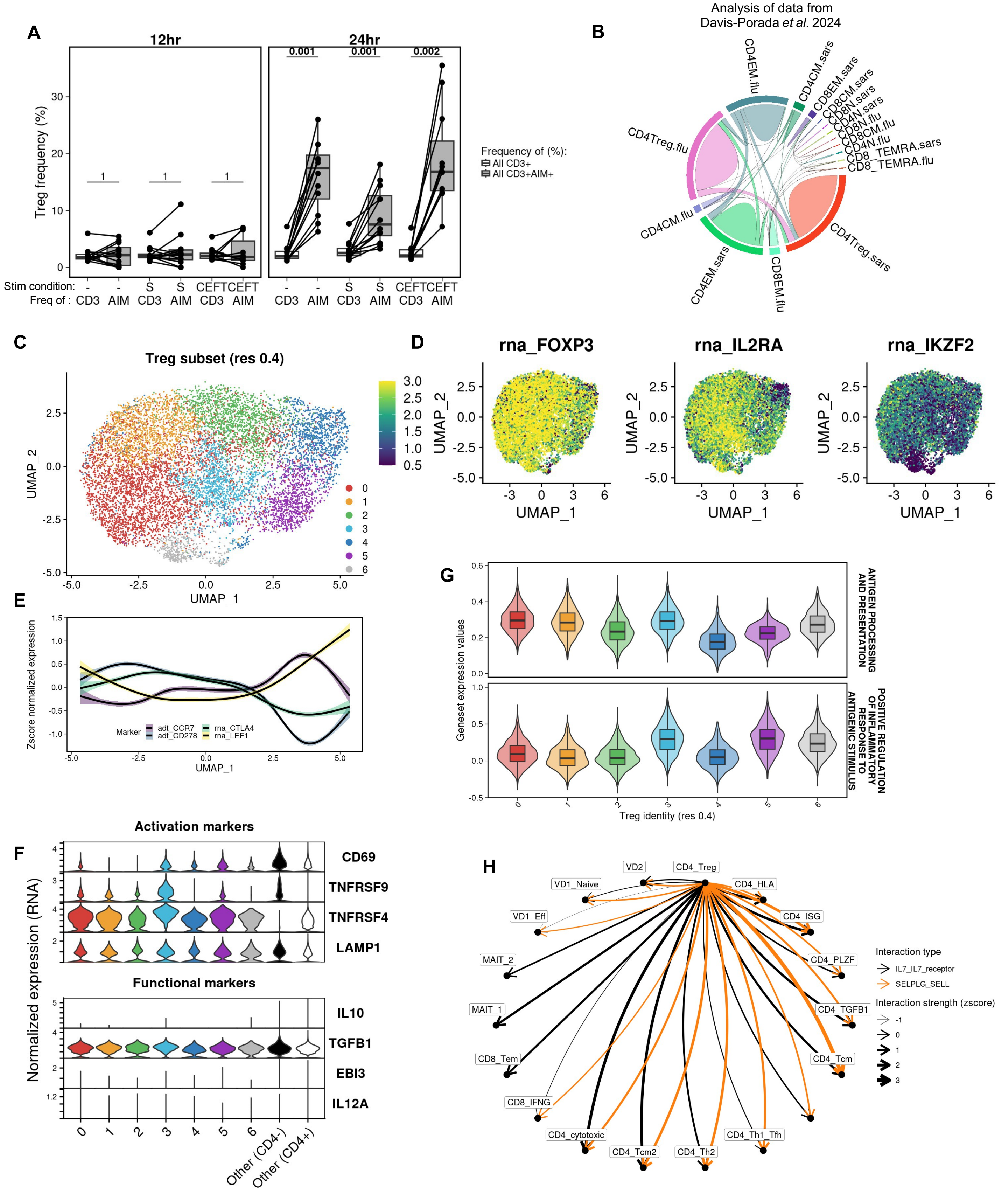

Fig. S9) A) Frequency of Treg cells in total CD3⁺ or AIM⁺ CD3⁺ T cells after 12- or 24-hour incubation with DMSO (-), SARS-CoV-2 spike peptides (S) or peptide pools covering immunodominant CD4⁺ T cell epitopes from cytomegalovirus, Epstein Barr virus, influenza virus and tetanus toxin (CEFT). P values are Benjamini-Hochberg false-discovery rates (FDR) from paired Mann-Whitney U tests **B)** Clonal overlap of TCRs derived from the peripheral blood of AIM⁺ T cells from mRNA vaccinated individuals in Davis-Porada *et al.* 2024 (32) (**Methods**). **C)** UMAP and Louvain clustering of CD4_Treg subset. **D)** Scaled expression of Treg associated genes. **E)** Scaled surface protein or gene expression of markers associated with effector and memory Tregs. **F)** Expression of activation induced marker and Treg functionality associated genes in Treg clusters and other CD4⁺ and CD4⁻ spike-responsive T cells. **G)** Aggregate gene expression for gene ontology biological pathway (GOBP) modules **H)** Selected unique or enriched CellphoneDB derived significant interactions made from Tregs to other spike-responsive T cell clusters.

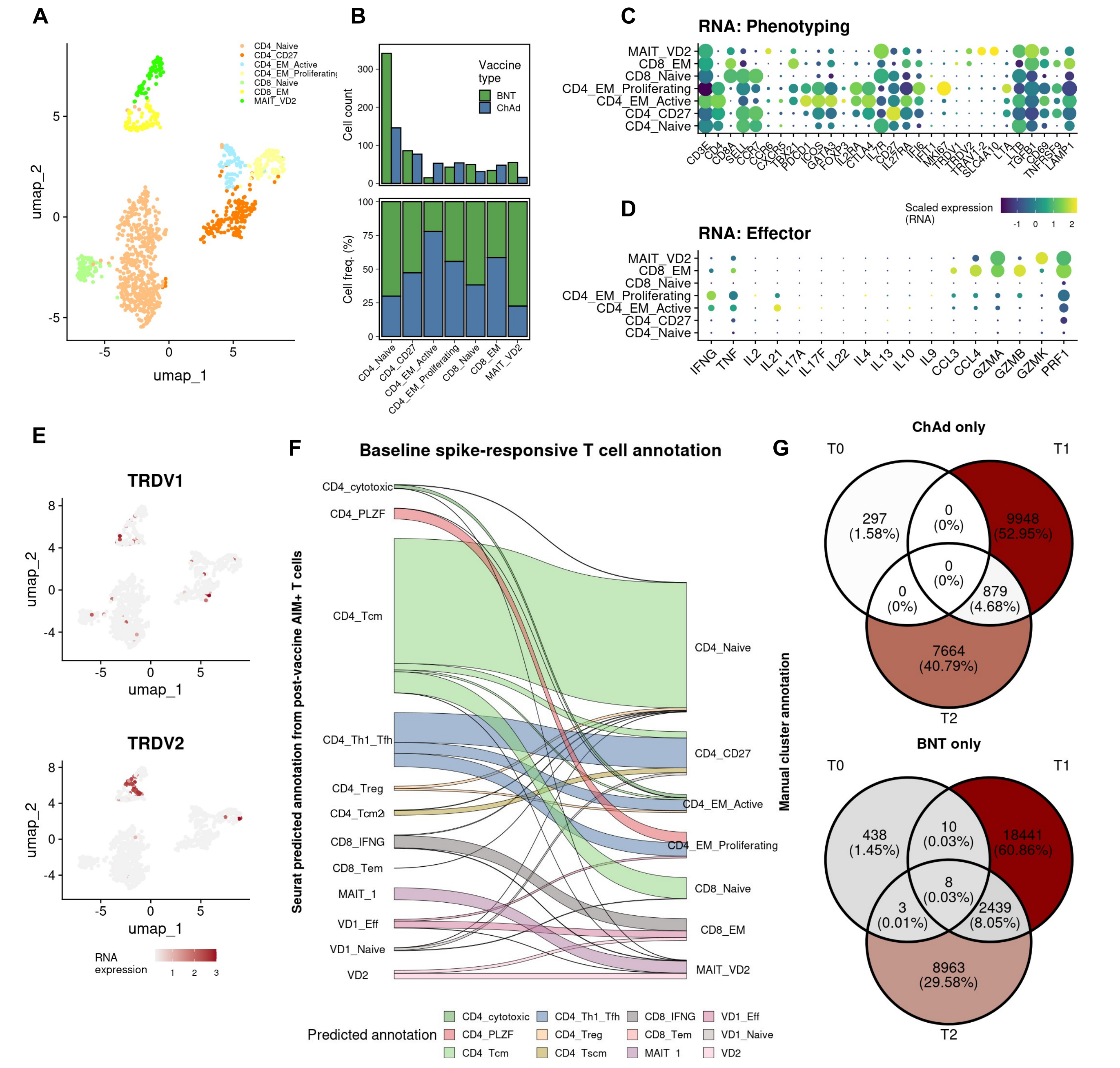

Fig. S10) Analysis of pre-vaccination spike-responsive CD3⁺ T cells detected using cell-trace violet assay. **A)** UMAP embedding of pre-vaccination spike-responsive T cells. **B)** Proportion of cells from each vaccination group in each pre-vaccination T cell cluster. **C and D)** Scaled gene expression of phenotyping (C) and effector (D) genes in pre-vaccination spike-responsive T cells. **E)** Scaled expression of TRDV1/2 genes. **F)** Comparison of manual annotation of pre-vaccination spike-responsive T cells with annotations mapped onto pre-vaccination spike-responsive T cell dataset from post- vaccination AIM⁺ spike-responsive T cells. **G)** Number of unique paired TCR clones that are shared across study timepoints in conventional spike-responsive T cells, split by vaccine type.

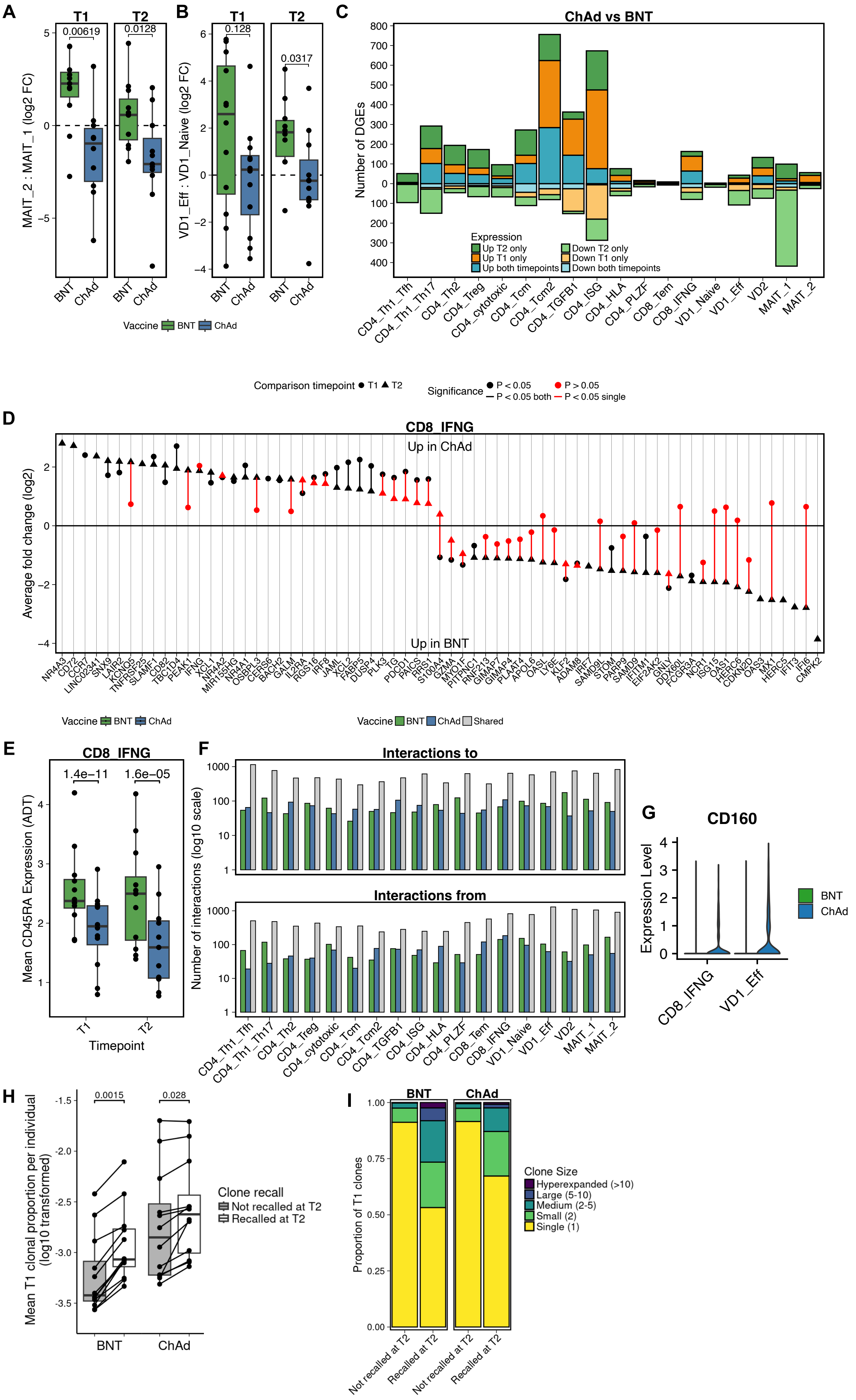
A
T1
T2
0.00619
0.0128
MAIT_2 : MAIT_1 (log2 FC)
BNT
ChAd
B
0.128
0.0317
VD1_Eff : VD1_Naive (log2 FC)
Vaccine BNT ChAd
C
ChAd vs BNT
Number of DGEs
Expression
Up T2 only
Up T1 only
Up both timepoints
Down T2 only
Down T1 only
Down both timepoints
CD4_Th1_Tfh
CD4_Th1_Th17
CD4_Th2
CD4_Treg
CD4_cytotoxic
CD4_Tcm
CD4_Tcm2
CD4_TGFB1
CD4_ISG
CD4_HLA
CD4_PLZF
CD8_Tem
CD8_IFNG
VD1_Naive
VD1_Eff
VD2
MAIT_1
MAIT_2
Comparison timepoint T1 T2
Significance P < 0.05 P > 0.05
P < 0.05 both P < 0.05 single
D
CD8_IFNG
Up in ChAd
Up in BNT
Average fold change (log2)
NR4A3
CD72
CCR7
LINC02341
SNX9
LAIR2
KCNQ5
TNFRSF25
SLAMF1
CD82
TBC1D4
PEAK1
IFNG
XCL1
NR4A2
MIR155HG
NR4A1
OSBPL3
CERS6
BACH2
GALM
IL2RA
RGS16
IRF8
JAML
XCL2
FABP5
DUSP4
PLK3
TG
PDCD1
PAICS
RRS1
S100A4
GZMA
MYO1F
PITPNC1
RNF213
GIMAP7
GIMAP4
PLAAT4
APOL6
OASL
LY6E
KLF2
ADAM8
IRF7
SAMD9L
STOM
PARP9
SAMD9
IFITM1
EIF2AK2
GNLY
DDX60L
FCGR3A
NCR1
ISG15
OAS1
HERC6
CDKN2D
OAS3
MX1
HERC5
IFIT3
IFI6
CMPK2
Vaccine BNT ChAd
Vaccine BNT ChAd Shared
E
CD8_IFNG
1.4e−11
1.6e−05
Mean CD45RA Expression (ADT)
Timepoint
F
Interactions to
Interactions from
Number of interactions (log10 scale)
G
CD160
Expression Level
BNT
ChAd
H
0.0015
0.028
Mean T1 clonal proportion per individual (log10 transformed)
Clone recall
Not recalled at T2
Recalled at T2
I
Proportion of T1 clones
Clone Size
Hyperexpanded (>10)
Large (5-10)
Medium (2-5)
Small (2)
Single (1)

Fig. S11) A and B) Log_2 ratio of cell frequencies of MAIT_1 to MAIT_2 (A) and VD1_Naive to VD1_Eff (B) (Wilcox signed-rank test, Bonferroni adjusted). **C)** Number of significantly differentially expressed genes (with log2 fold change > 0.25 or <-0.25) between ChAd and BNT vaccinees at T1 and T2 timepoints. **D)** Top 40 most significantly up and down regulated genes between ChAd and BNT vaccinees at T1 and T2 in the CD8_IFNG cluster. **E)** Mean per person CD45RA surface protein normalized expression in the CD8_IFNG cluster. **F)** Number of significant CellphoneDB derived interactions to the given cell type that are unique in spike-responsive T cells derived from a given vaccine type. **G)** Scaled gene expression of CD160 in selected spike-responsive T cell subsets. **H)** Mean proportion of T1 clones per donor that are recalled or not recalled at T2 (Mann-Whitney U-test, Bonferroni adjusted). **I)** Proportion of T1 clones of given clone size, split based on clones that are recalled at T2 and those that aren’t. Clones are called on a per donor basis.

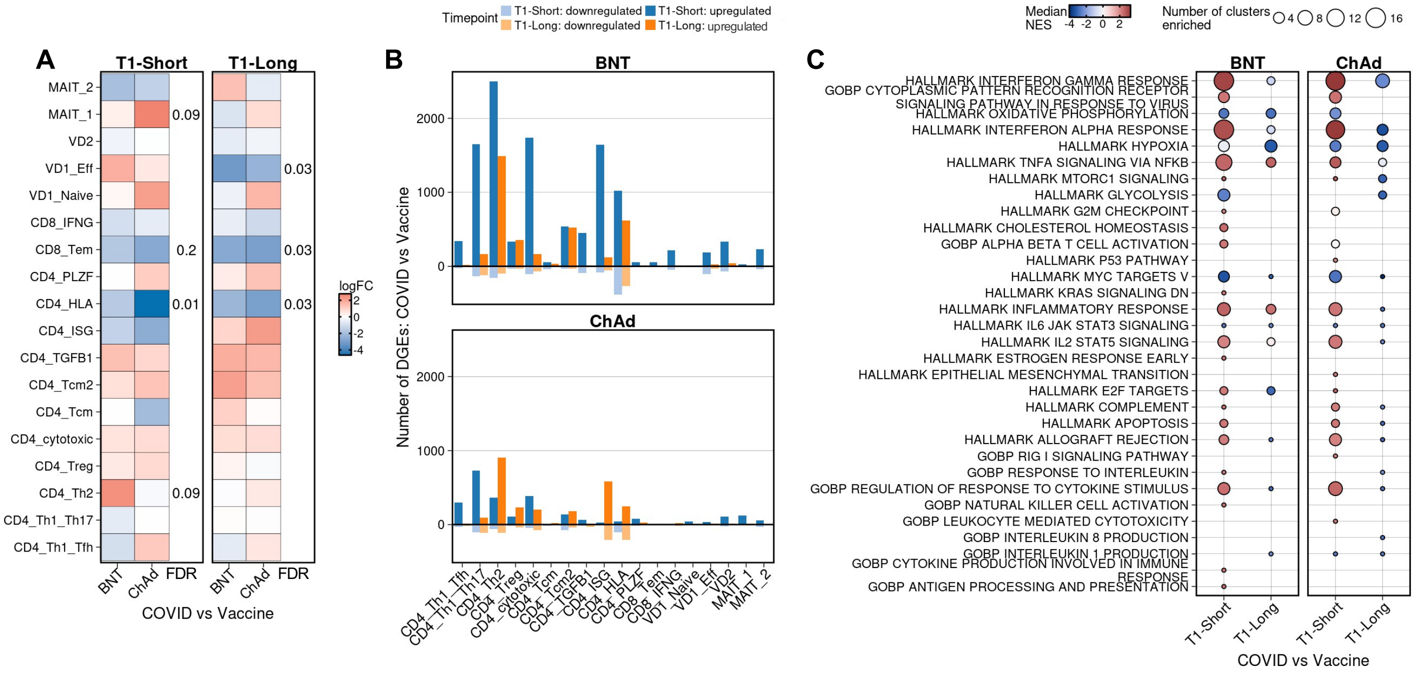

Fig. S12) A) Differential abundance analysis of COVID with vaccines at T1-short (early) and T1-long (late) sampling timepoints. False discovery rate (FDR) next to each cell type represents adjusted P of comparison at either timepoint (EdgeR F-test). **B)** Number of differentially expressed genes with average $\log_2$ fold change >0.25 in spike-responsive T cells at T1-short and T1-long timepoints in COVID compared to vaccinees. **C)** Geneset enrichment analysis of differentially expressed genes in COVID compared to vaccinees.

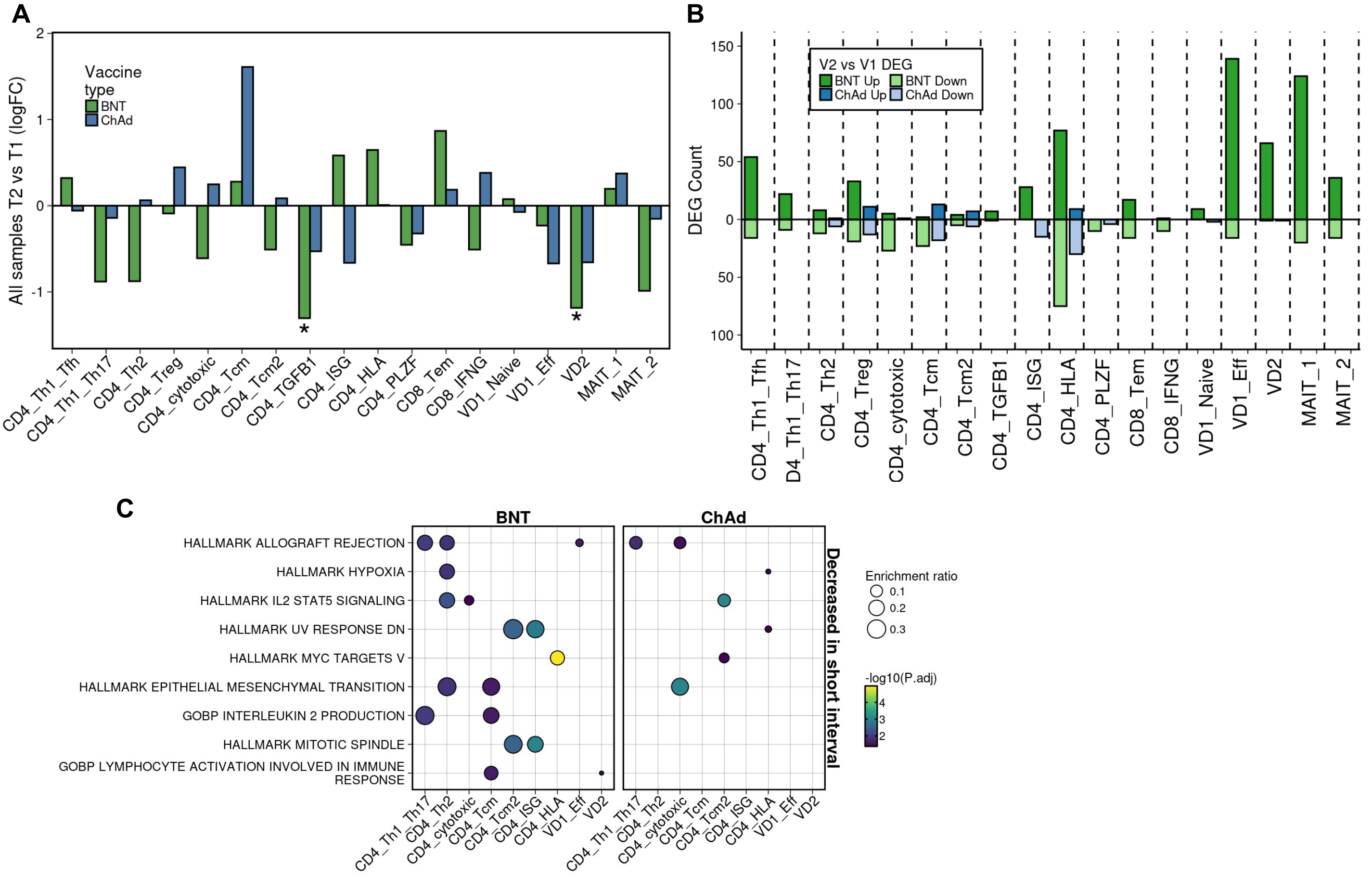

Fig. S13) A) Log_2 fold change of cell type frequencies at timepoint T2 compared to T1. FDR < 0.05 is marked with an *. **B)** Number of differentially expressed genes with average log_2 fold change >0.25 in spike-responsive T cells. **C)** Overrepresentation of sets of genes in genes that were downregulated in short compared to long interval vaccination.

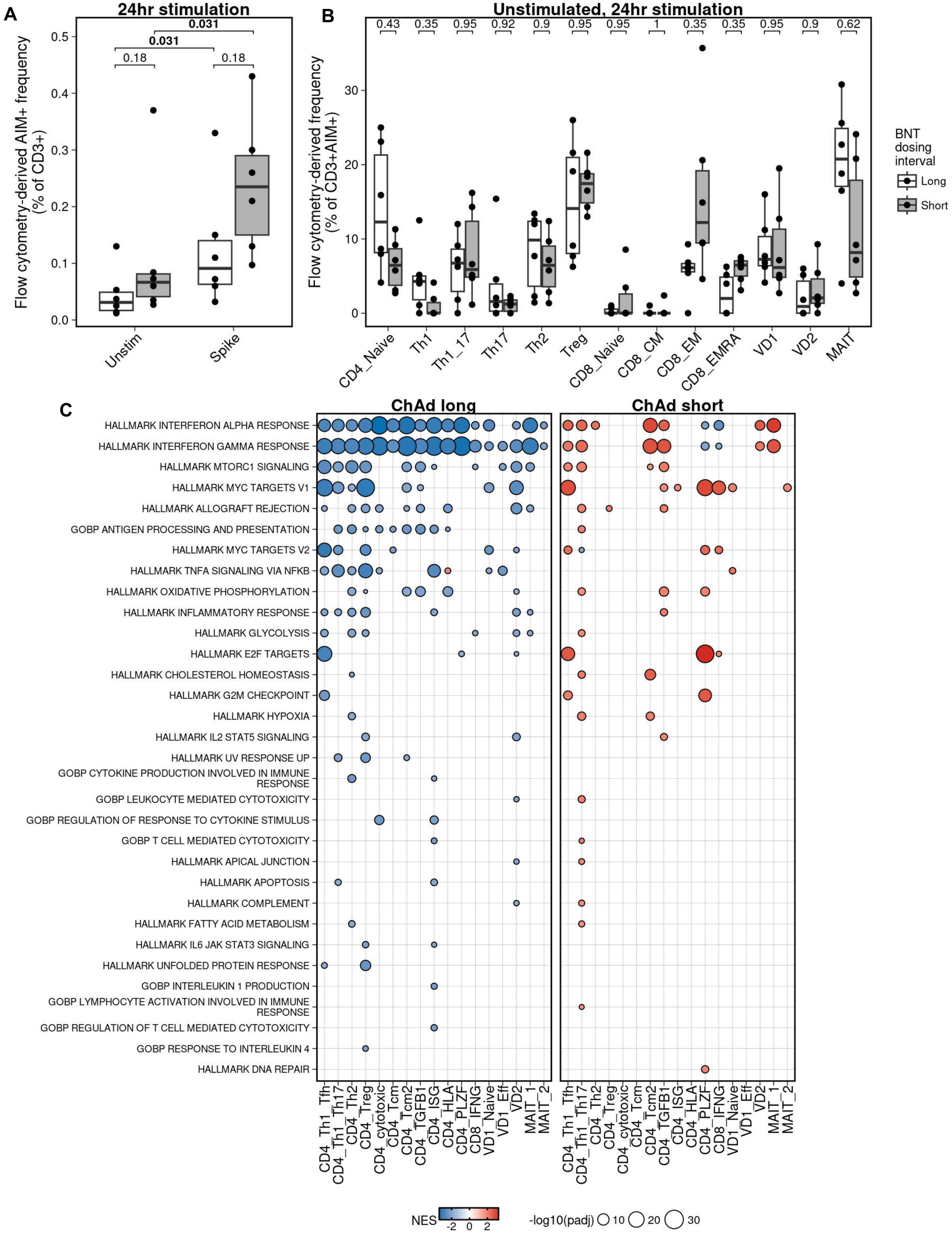

Fig. S14) A) Comparison of AIM⁺ CD3⁺ T cell populations in an independent flow cytometry experiment. T1 timepoint in n=12 individuals in BNT group. **B)** Frequency of T cell subsets in unstimulated AIM⁺ CD3⁺ T cells from flow cytometry experiment in A. **C)** Enrichment of genesets in genes ranked based on the strength association of their expression with the given comparison (**Methods**). Comparisons are made between paired timepoints, controlling for variation across individuals. Only enrichments with adjusted P value < 0.01 are shown.

Characteristic	BNT		ChAd			
	**l_BNT**, N = 6[1]	**s_BNT**, N = 6[1]	**l_ChAd**, N = 6[1]	**s_ChAd**, N = 6[1]	**COVID**, N = 6[1]	**p-value**[2]
Age	38 (33, 45)	42 (35, 52)	26 (22, 34)	30 (26, 34)	36 (31, 37)	0.10
Sex						>0.9
F	4 (67%)	3 (50%)	4 (67%)	3 (50%)	2 (33%)	
M	2 (33%)	3 (50%)	2 (33%)	3 (50%)	4 (67%)	

[1] Median (IQR); n (%)

[2] Kruskal-Wallis rank sum test; Fisher's exact test

Table. S1) Study group demographics

Interaction pair	Vaccine	No. interacting cell pairs	Interaction from	Interaction to
Adenosine_byNT5E_and_SLC29A1_ADORA2B	ChAd	1	CD4_Th1_Tfh	VD1_Naive
Adenosine_byNT5E_and_SLC29A2_ADORA2A	ChAd	2	CD4_Th1_Tfh	VD1_Naive, MAIT_2
Adenosine_byNT5E_and_SLC29A3_ADORA2A	ChAd	2	CD4_Th1_Tfh	VD1_Naive, MAIT_2
CD160_TNFRSF14	ChAd	18	CD8_IFNG	VD1_Naive, MAIT_2, CD4_HLA, CD4_PLZF, CD4_ISG, CD4_TGFB1, CD4_Tcm, CD4_Th1_Th17, CD4_Th1_Tfh, CD4_Th2, CD4_Treg, CD4_Tscm, CD4_cytotoxic, CD8_IFNG, CD8_Tem, MAIT_1, VD1_Eff, VD2
CD160_TNFRSF14	ChAd	18	VD1_Eff	VD1_Naive, MAIT_2, CD4_HLA, CD4_PLZF, CD4_ISG, CD4_TGFB1, CD4_Tcm, CD4_Th1_Th17, CD4_Th1_Tfh, CD4_Th2, CD4_Treg, CD4_Tscm, CD4_cytotoxic, CD8_IFNG, CD8_Tem, MAIT_1, VD1_Eff, VD2
CD248_IL6R	BNT	1	VD1_Naive	CD4_Treg
CD80_CD28	ChAd	1	CD4_Th1_Th17	MAIT_1
CD80_CD28	ChAd	1	CD4_Treg	MAIT_1
DLL3_NOTCH1	BNT	1	CD4_Th1_Tfh	VD1_Eff
Dehydroepiandrosterone_bySTS_ESR1	ChAd	1	CD4_Th1_Tfh	CD4_PLZF
Dehydroepiandrosterone_bySTS_ESR1	ChAd	1	CD8_IFNG	CD4_PLZF
Dehydroepiandrosterone_bySTS_ESR1	ChAd	1	VD1_Naive	CD4_PLZF
EFNB1_EPHA4	BNT	1	CD4_Th1_Tfh	CD4_Tcm
ENTPD1_ADORA2B	ChAd	1	CD4_Treg	VD1_Naive
IL21_IL21_receptor	BNT	8	CD4_Th1_Tfh	VD1_Naive, MAIT_2, CD4_Th1_Tfh, CD4_Treg, CD8_IFNG, MAIT_1, VD1_Eff, VD2
L1CAM_integrin_a5b1_complex	ChAd	4	CD4_TGFB1	CD4_TGFB1, CD4_Th2, CD4_Treg, MAIT_1
L1CAM_integrin_aVb1_complex	ChAd	3	CD4_TGFB1	CD4_TGFB1, CD4_Th2, CD4_Treg
NCR3LG1_NCR3	ChAd	3	CD8_IFNG	MAIT_2, MAIT_1, VD2
NCR3LG1_NCR3	ChAd	3	VD1_Naive	MAIT_2, MAIT_1, VD2
ProstaglandinD2_byAKR1C3_PTGDR	BNT	1	VD2	CD8_IFNG
THBS1_integrin_a3b1_complex	ChAd	1	CD4_HLA	CD4_TGFB1
THBS1_integrin_a3b1_complex	ChAd	2	CD8_Tem	MAIT_2, CD4_TGFB1
THBS1_integrin_a3b1_complex	ChAd	1	MAIT_1	CD4_TGFB1
TNFSF9_TNFRSF9	ChAd	5	CD8_IFNG	VD1_Naive, CD4_Th1_Tfh, CD8_IFNG, MAIT_1, VD1_Eff
ULBP2_NKG2D_II_receptor	ChAd	7	CD4_Th1_Th17	VD1_Naive, MAIT_2, CD8_IFNG, CD8_Tem, MAIT_1, VD1_Eff, VD2

Table. S2) Significant CellphoneDB derived interactions which are unique to the given vaccine type, with breakdown of the type of interaction and the uniquely interacting cell types.
